# The origin and evolution of methanogenesis and *Archaea* are intertwined

**DOI:** 10.1093/pnasnexus/pgad023

**Published:** 2023-01-31

**Authors:** Ran Mei, Masanori Kaneko, Hiroyuki Imachi, Masaru K Nobu

**Affiliations:** Bioproduction Research Institute, National Institute of Advanced Industrial Science and Technology (AIST), 1-1-1 Higashi, Tsukuba 305-8566, Japan; Institute for Geo-Resources and Environment, Geological Survey of Japan, National Institute of Advanced Industrial Science and Technology (AIST), 1-1-1 Higashi, Tsukuba 305-8567, Japan; Institute for Extra-cutting-edge Science and Technology Avant-garde Research (X-star), Japan Agency for Marine-Earth Science and Technology (JAMSTEC), 2-15 Natsushima-cho, Yokosuka, Kanagawa 237-0061, Japan; Bioproduction Research Institute, National Institute of Advanced Industrial Science and Technology (AIST), 1-1-1 Higashi, Tsukuba 305-8566, Japan; Institute for Extra-cutting-edge Science and Technology Avant-garde Research (X-star), Japan Agency for Marine-Earth Science and Technology (JAMSTEC), 2-15 Natsushima-cho, Yokosuka, Kanagawa 237-0061, Japan

**Keywords:** evolution, methanogenesis, *Archaea*

## Abstract

Methanogenesis has been widely accepted as an ancient metabolism, but the precise evolutionary trajectory remains hotly debated. Disparate theories exist regarding its emergence time, ancestral form, and relationship with homologous metabolisms. Here, we report the phylogenies of anabolism-involved proteins responsible for cofactor biosynthesis, providing new evidence for the antiquity of methanogenesis. Revisiting the phylogenies of key catabolism-involved proteins further suggests that the last *Archaea* common ancestor (LACA) was capable of versatile H_2_-, CO_2_-, and methanol-utilizing methanogenesis. Based on phylogenetic analyses of the methyl/alkyl-S-CoM reductase family, we propose that, in contrast to current paradigms, substrate-specific functions emerged through parallel evolution traced back to a nonspecific ancestor, which likely originated from protein-free reactions as predicted from autocatalytic experiments using cofactor F_430_. After LACA, inheritance/loss/innovation centered around methanogenic lithoautotrophy coincided with ancient lifestyle divergence, which is clearly reflected by genomically predicted physiologies of extant archaea. Thus, methanogenesis is not only a hallmark metabolism of *Archaea*, but the key to resolve the enigmatic lifestyle that ancestral archaea took and the transition that led to physiologies prominent today.

**Significance Statement**

How each domain of life emerged on ancient Earth and evolved modern diversity is a longstanding mystery. For *Archaea*, biological methane production (“methanogenesis”), an ability unique to the domain, is believed to be primitive, but how it originated, evolved, and contributed to the domain's development remains hotly debated. Based on phylogenetic and experimental analyses, we found new evidence that methane (and other alkanes) metabolism preceded the origin of *Archaea* and the innovation of a protein dedicated to methane production coincided with the emergence of LACA. From this ancestor, downstream inheritance and loss of methane metabolism paralleled early diversification of the domain, pointing toward a key role of methanogenesis in the origin and evolution of *Archaea*.

## Introduction

Our understanding of archaeal diversity on modern Earth is rapidly expanding (1), providing more opportunities to address the enigmatic questions of how the archaeal ancestor originated and diversified to extant forms. Methanogenesis, a feature exclusive to the domain, is one of the few catabolisms that could sustain primordial life by taking advantage of gases abundant then, H_2_ for energy and CO_2_ for cell carbon. This lithoautotrophic way of life could have also profoundly influenced the emergence and/or proliferation of other lifestyles by providing primary production and lifting thermodynamic restrictions for organoheterotrophy (i.e. via H_2_ consumption) (2). Thus, it is tempting to suspect that methanogenesis is not only an ancient metabolism but intricately intertwined with the origin and diversification of *Archaea*—the trajectory of early evolution cannot be comprehended without investigating the two in parallel.

The notion that methanogenesis is ancient has been widely accepted (1, 3, 4) since it was first proposed in the 1970s (5, 6), yet several key questions remain unresolved (see details in Supplementary Information), blurring our understanding of the evolution of *Archaea*, methanogenesis, and their relationship. The origin of methanogenesis was historically placed within Euryarchaeota (7, 8). With recent discoveries of methanogenic lineages in the TACK superphylum (an assemblage of archaeal phyla that includes Thaumarchaeota, Crenarchaeota, Korarchaeota, and Aigarchaeota) (9–12), the origin has been moved further down the archaeal tree to the last common ancestor (LCA) of Euryarchaeota and TACK. However, whether methanogenesis can be traced earlier remains unexplored with molecular phylogeny. Moreover, discoveries of divergent homologs of the central enzyme of methanogenesis, methyl-*S*-CoM reductase (Mcr), that activate nonmethane alkanes (13–20) (e.g. Ecr: ethyl-*S*-CoM reductase and Acr: alkyl-*S*-CoM reductase) has raised discussions about the history of the entire protein family (12, 13, 16, 21) but with no clear conclusion. As for the original form of methanogenesis, whether methylated compounds or CO_2_ was the starting compound remains debated (9, 21). In addition, while several studies have revealed the evolution and physiology of archaeal lineages that lost methanogenesis (22–24), an explicit demonstration of how the inheritance/loss/gain/innovation of methanogenesis drove early evolution at the entire domain scale is lacking.

In this study, we integrated phylogenetic investigations of both catabolism- and anabolism-involved proteins related to methanogenesis to provide new insights to the elusive evolutionary history mentioned above. Catabolism-involved proteins are generally more directly influenced by the dynamic extracellular milieu (e.g. availability and diversity of energy sources) and, thus, could better record complex functional diversification, whereas anabolism-involved proteins tend to reflect history of vertical evolution. By analyzing the history of genes hitherto unexplored (in terms of phylogeny) and revisiting those under debate, examining the catalytic activity of a key cofactor, and performing a domain-wide genome-based prediction of physiologies, we aim to give a new projection of how methanogenesis itself evolved and, more importantly, how it potentially drove the origination and initial diversification of the domain *Archaea*.

## Results and discussion

### Tracing catabolic and anabolic genes for methanogenesis to last *Archaea* common ancestor

**Fig. 1.**
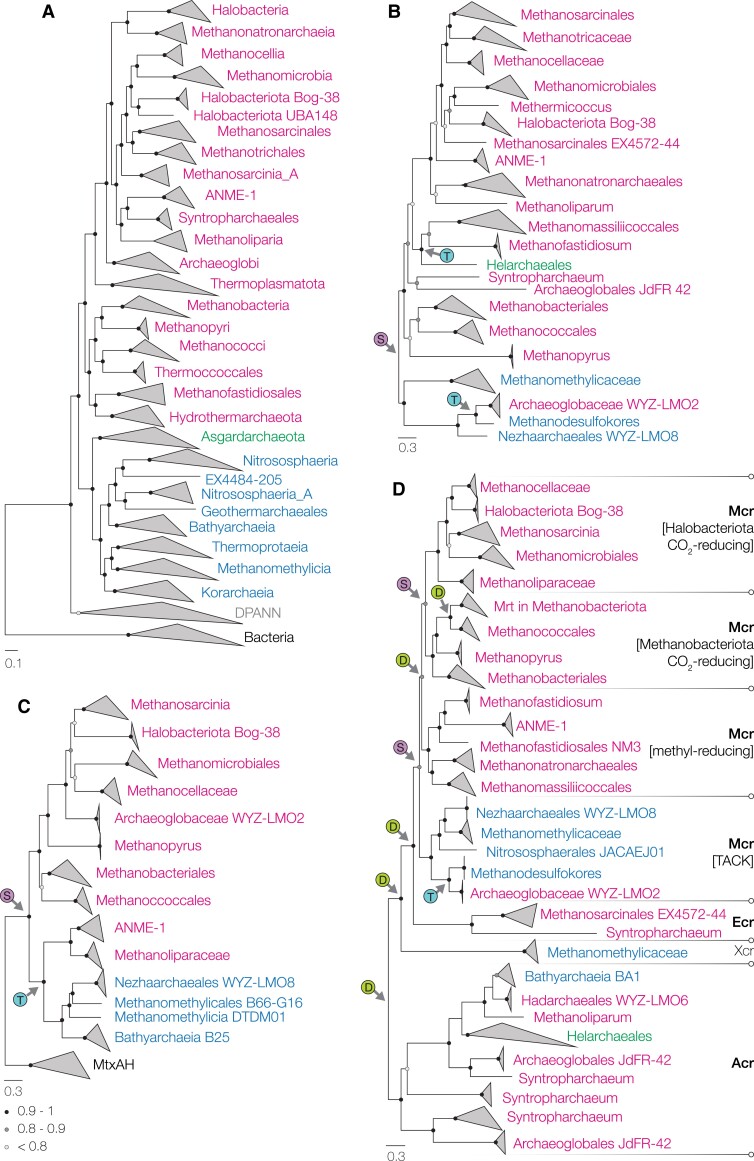
Phylogeny of (A) *Archaea*, (B) CfbABCDE, (C) MtrAH, and (D) McrBDCGA. The archaeal species tree in (A) was constructed using the maximum-likelihood method. Taxa are colored according to Euryarchaeota (pink), TACK (blue), Asgardarchaeota (green), and DPANN (gray). It is collapsed at various levels to highlight lineages related to methanogenesis (uncollapsed version in Fig. S1). Circles at nodes denote tbe-transferred ultrafast bootstrap supports. Trees in (B–D) were constructed based on Bayesian inference. Circles at nodes denote posterior probabilities. Key evolutionary events (S for speciation, D for duplication, and T for horizontal transfer) that are mentioned in the main text are labeled. The same trees with expanded clades and more labels are shown in Figs. S2, S17, and S28, respectively.

Five anabolism-involved proteins (CfbABCDE) constitute the biosynthesis pathway of cofactor F_430_, the indispensable coenzyme for methanogenesis (25, 26). We searched public genome database (27) and found the complete pathway in most genomes that contain Mcr or Acr, consistent with a previous report (13). No genome contains duplicate Cfb pathway, even those with multiple copies of Mcr and Acr (e.g. Syntropharchaeum and Methanoliparium). Highly congruent with the topology of the archaeal species tree (Fig. 1A; full version with uncollapsed nodes in Fig. S1), phylogeny of the concatenated alignment of CfbABCDE revealed a basal divergence between TACK and Euryarchaeota (Figs. 1B and S2), supported by the phylogenies of individual Cfb proteins that are rooted at functionally related bacterial homologs (Figs. S3–S7). Reconciling the CfbABCDE gene tree against the species tree further confirmed this basal divergence and revealed that this divergence resulted from a speciation event instead of horizontal gene transfer (Fig. 1B). Clear horizontal transfers were found at shallow positions in the tree such as Archaeoglobi WYZ-LMO2 and Helarchaeales. Taken together, we could trace F_430_ biosynthesis to, at the latest, the divergence of TACK and Euryarchaeota, providing new support for the antiquity of methanogenesis. Based on the archaeal species trees constructed here (Figs. S1 and S8) and reported elsewhere (Supplementary Information), it is tempting to conclude that F_430_ biosynthesis and, by extension, methanogenesis was present at last *Archaea* common ancestor (LACA).

What type of methanogenesis LACA was metabolically capable of (i.e. CO_2_- vs. methyl-reducing) remains controversial (3, 9, 13, 21, 24). Methyl-reducing methanogenesis has been suggested to be ancient based on the antiquity of methylcobamide:CoM methyltransferase (MtaA) (21). We find consistent results that both MtaA (Fig. S9) and the upstream methanol-interacting methylcobamide-generating methyltransferase MtaB (Fig. S10) originated prior to the divergence of TACK and Euryarchaeota, suggesting that LACA was capable of methanogenic reduction of methanol, a compound that could be abiotically generated in primordial hydrothermal vent conditions (28). On the other hand, the key protein for CO_2_-reducing methanogenesis, methyl-tetrahydromethanopterin methyltransferase (Mtr), was suggested to be nearly exclusive to Euryarchaeota (with only one lineage detected in TACK) and has an later origin (21). Here, we find the complete mtrEDCBAFGH gene operon in another three TACK orders, signifying an underestimated distribution. In the phylogenies of individual subunits (E/D/C/B/A/H, Figs. S11–S16; mtrF and mtrG are omitted because they are both very short and are subject to frequent gene fusion (29)), we observed a basal divergence between TACK and Euryarchaeota, supported by gene–species reconciliation. The topology between the gene trees and species tree is congruent for all but two lineages—Methanoliparia and ANME-1, the only two lineages capable of alkane oxidation in the Mtr trees. These two lineages always form a robust subcluster, sometimes branching next to TACK (as in the phylogenies of mtrA/B/E/H) but occasionally grouping sister to Methanocellia (in mtrC) or within Methanobacteriota (in mtrD), suggesting that their mtr genes were not vertically inherited but rather acquired horizontally through a complex history. The deep divergence between TACK (and the associated Methanoliparia/ANME-1) and Euryarchaeota is also observed in the phylogeny of the concatenated catalytic subunits MtrAH (Figs. 1C and S17), which could be rooted with the corresponding subunits of a homologous complex with a distinct function (MtxAH) (30). Reconciliation analysis reveals that MtrAH was present at the TACK-Euryarchaeota LCA. The Methanoliparia/ANME-1 subcluster was again predicted to have resulted from a horizontal transfer from a deep-branching TACK lineage that might be unsampled or extinct. The TACK-Euryarchaeota division and horizontal transfer to Methanoliparia/ANME-1 are further supported by the unique operon structure of Mtr (EDCB[AF]FGH, with mtrA fused with a duplicate mtrF) observed in the TACK clade (and the associated Methanoliparia and ANME-1; Fig. S17). Overall, Mtr in TACK likely resulted from a basal divergence from Euryarchaeota rather than evolving from with Euryarchaeota, and thus, Mtr was present at least at the divergence of TACK and Euryarchaeota.

In addition to Mtr, CO_2_-reducing methanogenesis requires the methyl-branch of the Wood–Ljungdahl pathway (WLP), which has been reported to be ancient in *Archaea* (31, 32). In line with the reports based on catabolism-involved proteins, we observed consistent division between TACK (with Asgardarchaeota nested together) and Euryarchaeota in the phylogenies of anabolism-involved proteins for the biosynthesis of CO_2_-reduction-essential cofactors (methanofuran, molybdopterin, methanopterin, and coenzyme F_420_; Figs. S18–S21), corroborating the antiquity of CO_2_ reduction. In addition, we found evidence that LACA could likely link CO_2_-reducing methanogenesis and H_2_ lithotrophy (vertical inheritance of proteins responsible for [NiFe] hydrogenase maturation, Fig. S22; F_420_-mediated electron transfer from H_2_ to CO_2_, Fig. S23), autotrophy (carbon monoxide dehydrogenase/acetyl-CoA synthase; Fig. S24), and the tricarboxylic acid cycle (CoM-S-S-CoB thiol:fumarate reductase; Fig. S25), suggesting a central role of lithoautotrophic methanogenesis in LACA's physiology (though LACA may have been capable of other additional lifestyles, e.g. mixotrophy) (33).

### Elucidate the evolution of the methyl/alkyl-*S*-CoM reductase protein family

How far back can we trace the innovation of methanogenesis? How does this innovation relate to the origin of *Archaea*? Here we capitalize on the recently expanded Mcr protein family (MCRpf, e.g. Ecr and Acr interacting with different alkanes) to explore the history of archaeal methane/alkane metabolism. The evolutionary trajectory of this protein family has been discussed (12, 13, 16, 21), but whether the different functions of the family evolved in parallel as distinct lineages or through neofunctionalization (i.e. one function evolved from within another) remains unknown. Without resolving this, the evolution of the function cannot be related to the evolution of the domain (as done above for other functions). In the phylogeny of the three catabolism-involved proteins that constitute the Mcr/Acr protein complex (BGA, Fig. S26), gene–species reconciliation and minimum ancestor deviation both reveal that the basal divergence is placed at Acr and that MCRpf homologs clearly separate according to the alkane they interact with, a topology suggestive of parallel evolution. However, such phylogenetic separation could be claimed as a tree reconstruction artifact due to high sequence divergence following a shift in function, i.e. EcrBGA and AcrBGA actually emerged from within McrBGA but form distant clades in a phylogenetic tree (13). To address this, we analyzed the phylogenies of two anabolism-involved proteins that support the function of BGA complexes: protein C for enzyme activation (34) and D for post-translational assembly (35). Proteins C/D are ideal targets for differentiating parallel evolution and neofunctionalization because their association with AcrBGA or McrBGA is explicit (Figs. S27 and S28, and details in the figure caption) but the phylogeny ought to be not directly influenced by the target alkane as they (i) are anabolism-involved proteins that do not interact with the substrates and (ii) can be compatible with BGA complexes targeting different alkanes, as evidenced by the presence of single copies of C/D in genomes with multiple copies of BGA. Gene–species reconciliation of a phylogenetic tree based on a concatenated alignment of proteins C and D showed a basal divergence between those associated with Acr and those associated with other MCRpf homologs (Fig. S27). This shows that the divergence between AcrCD and McrCD, whose functions are not influenced by substrate type, is not caused by neofunctionalization toward different alkanes and, by extension, the divergence between AcrBGA and McrBGA is also not caused by neofunctionalization.

Given the similar topology of proteins BGA and CD, we constructed a phylogenetic tree using a concatenated alignment of the five proteins to increase the resolution of the analysis (Figs. 1D and S28). Gene–species reconciliation of the BGACD tree recovered the same topology and rooting as BGA and CD and further projected that (i) Acr, Xcr (a deep clade exclusively composed of Methanomethylicaceae), Ecr, and Mcr evolved through duplications and (ii) the LCA of Mcr was present at the LCA of TACK-Euryarchaeota. This not only verifies that methanogenesis was available prior to the TACK-Euryarchaeota divergence (i.e. likely at LACA), but also indicates that those duplications toward different MCRpf functions were more ancient. For Mcr, after the divergence of TACK and Euryarchaeota is a duplication within Euryarchaeota leading to the methyl- and CO_2_-reducing Mcr. We further observed a clear transition in operon structure at the emergence of Mcr from transcriptionally discrete BGA and CD in all non-Mcr MCRpf clusters to a conserved BDCGA operon that is unique to and ancestral among Mcr (Fig. S28). The integration of CD into the same transcriptional unit as BGA may reflect a potentially critical specialization to a specialized MCRpf function (methanogenesis, in this case), giving rise to a bona fide methanogenic archaeon at LACA.

The abovementioned inference places methane-specific Mcr as a relatively recent innovation within the entire protein family—does this imply that biological methanogenesis was not available until Mcr emerged at LACA? Given Mcr-, Ecr-, and Acr-possessing organisms conserve dependency on the same cofactor F_430_ and share a single F_430_ biosynthesis pathway, F_430_ likely predated the diversification of the protein family. We demonstrated that reduced F_430_ alone (i.e. in the absence of the apoenzyme) autocatalyzed methane production from methyl-*S*-CoM (Fig. S29), implying that F_430_ could mediate methanogenesis without, and likely prior to, the emergence of Mcr. This provides a concrete example for the theory that cofactors evolved during the transition from mineral-catalyzed surface metabolism toward protein-enveloped enzymes (36). The results that F_430_ can autocatalyze methane production from other methylated compounds (Fig. S29) and reductive dechlorination (37) reflect the catalytic versatility of F_430_ (i.e. compatible with all members of the MCRpf that interact with different substrates). Based on the results above, we further hypothesize that the ancestor of the MCRpf, owing to the versatility of F_430_, was a nonspecific reductase that could interact with a wide range of alkanes including methane, in accordance with the evolutionary phenomenon of enzyme promiscuity (i.e. ancestral enzymes have wider ranges of activities than their descendants) (38).

### Diversification of major archaeal lineages

**Fig. 2.**
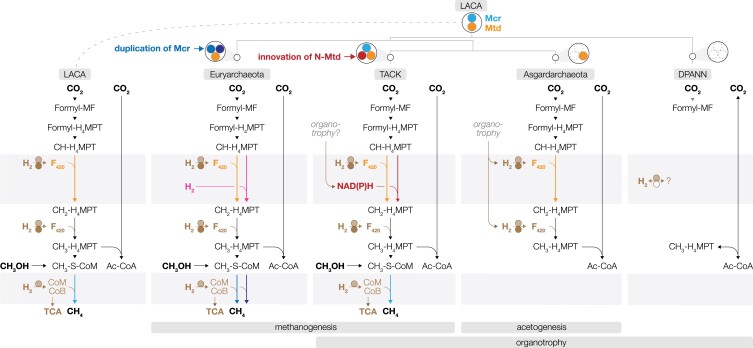
Ancestral physiology and lifestyle inferred from the presence/absence of WLP and methanogenesis at LACA and the last common ancestors of each major archaeal lineage. The presence of specific enzymes is highlighted with different colors. Orange for Mtd-mediated F_420_-H_2_-driven methylene-H_4_MTP reduction; red for *N*-Mtd-mediated NAD(P)H-driven methylene-H_4_MTP reduction; pink for Hmd-mediated H_2_-driven methylene-H_4_MTP reduction; light blue for LACA-like Mcr; dark blue for duplicated Mcr at Euryarchaeota; light brown for [NiFe] hydrogenase maturation complex; dark brown for F_420_-H_2_ reductase.

Starting from the methanogenic LACA, what were the physiological distinctions that underlay the divergence toward the four major archaeal lineages, i.e. Euryarchaeota, DPANN, TACK, and Asgardarchaeota? We discovered stark contrasts in how each major lineage inherited and modified methanogenesis and the associated WLP (Fig. 2). Each ancestor of TACK and Euryarchaeota inherited methanogenesis (Mcr, Mtr, Mta, and Cfb) from LACA, while each ancestor of DPANN and Asgardarchaeota lost these genes. Within Euryarchaeota, we further observed two duplications of Mcr—one dividing CO_2_- and methyl-reducing lineages and the other confined to Methanobacteriales. The former may have taken place to support the distinct thermodynamic properties of CO_2_- and methyl-reducing methanogenesis (i.e. 2 vs. 0.01 mM intracellular methyl-*S*-CoM concentrations) as predicted based on quasi-equilibrium calculations (Table S1). The latter is suggested to adapt to different thermodynamic conditions (Mcr I and II) (39). Such diversification of Mcr is not observed in TACK, though whether this is due to poor sampling of TACK methanogens or nonspecific activity of ancestral Mcr (inherited by TACK) remains unknown.

In parallel with methanogenesis, the ancestors of each major lineage evolved to utilize different forms of the WLP, with the distinction spotted at the methylene-H_4_MTP dehydrogenation step. It is the only known modification that archaea made on the WLP—using either F_420_, NAD(P), or H_2_ as the electron carrier. The phylogeny of the F_420_-dependent Mtd shows Euryarchaeota, Asgardarchaeota, and TACK members (no DPANN) vertically inherited Mtd from LACA (Fig. S30), consistent with previous reports (31). In contrast, NAD(P)H-dependent methylene-H_4_MPT dehydrogenase (N-Mtd) is detected in TACK, DPANN, and Asgardarchaeota but not Euryarchaeota (Fig. S31). The rooted N-Mtd tree shows that this is an archaeal innovation and, based on the basal position of TACK, absence in Euryarchaeota, nesting of DPANN and Asgardarchaeota in shallow positions of the tree, likely emerged near or within TACK (though the specific timing remains unclear). The last variance, the H_2_-dependent methylene-H_4_MPT dehydrogenase (Hmd) is confined to Methanopyri, Methanococci, and Methanobacteria (with horizontal transfer to some Methanomicrobia, Fig. S32), suggesting that this function was innovated late within Euryarchaeota. Clearly, there are differences in how the four major lineages utilize the WLP.

These results reveal that, when LACA diverged toward the four major lineages, methanogenesis and the WLP were inherited, lost, or modified (Fig. 2). While duplication of Mcr likely allowed the Euryarchaeota ancestor to develop lithoautotrophic methanogenesis adapted to specific conditions, N-Mtd in the other three lineages likely allowed them to link the WLP to NAD(P)-centric metabolism (i.e. organotrophy). The TACK ancestor retained most genes supporting LACA's core physiology (methanogenesis, most part of the WLP, H_2_ metabolism, and F_420_-H_2_ redox), but acquisition of N-Mtd indicates a lower dependency of WLP on H_2_ and divergence from the lithoautotrophic lifestyle LACA took. The Asgardarchaeota ancestor vertically inherited WLP, F_420_ biosynthesis, F_420_-H_2_ redox, Mtd, and [NiFe] hydrogenase maturation but lost methanogenesis, implying nonmethanogenic coupling of WLP and H_2_ oxidation. Taken together with a previous report of organotrophy as an ancestral (and extant) feature (40), we suspect that Asgardarchaeota performed H_2_-generating organotrophy and could support this with H_2_-consuming acetogenesis, common among cultured bacterial acetogens that (i) all possess organotrophic capacities (i.e. no exclusively lithotrophic acetogens reported to date), (ii) preferentially perform H_2_-generating organotrophy over acetogenesis (41), (iii) consume organotrophy-derived H_2_ to drive acetogenesis (i.e. internal H_2_ cycling or “syntrophy in one bacterial cell”) in the absence of methanogens (42), and (iv) cannot thermodynamically compete with methanogens for exogenous H_2_ (43). The DPANN ancestor experienced the most severe loss of lithoautotrophic capacities as evidenced by the absence of a complete archaeal WLP and F_420_-H_2_ redox.

**Fig. 3.**
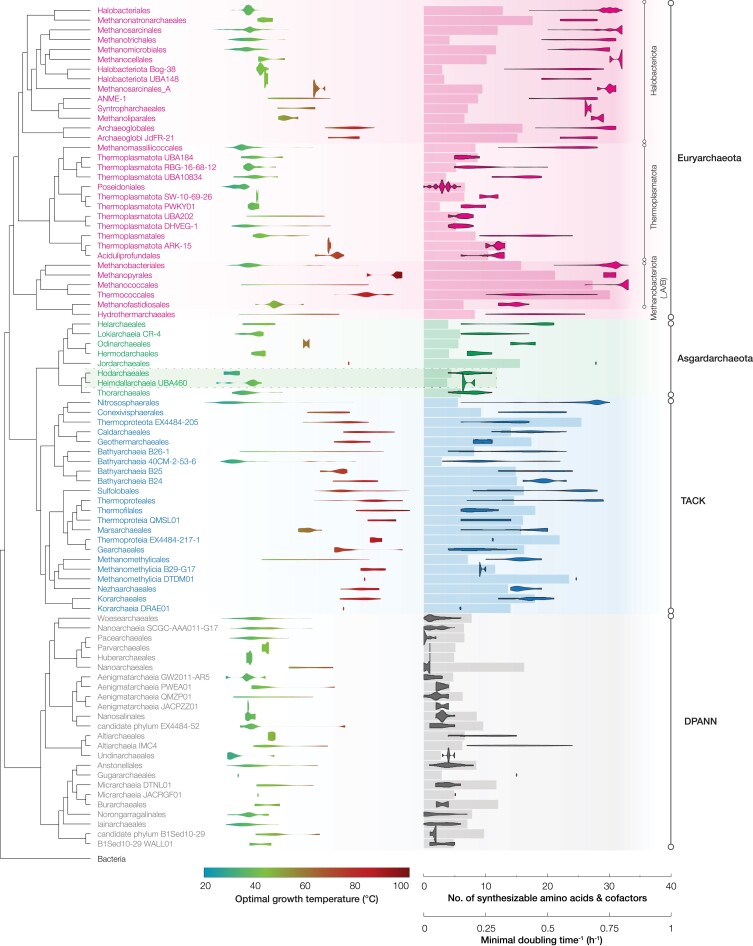
Transition of growth characteristics along the loss of methanogenesis. Cladogram is a maximum-likelihood genome tree collapsed at order level (uncollapsed version in Fig. S1). The violin plot on the left shows the optimal growth temperature of each order and the one on the right shows the biosynthetic capacity (number of amino acids and cofactors that could be synthesized). The bar plot shows the growth rate (average reciprocal of minimal doubling time) of each order. Heimdallarchaeia, the closest relative to Eukarya's archaeal ancestor reported to date, is highlighted.

We further observed trends in the extant physiologies of the four major lineages that coincide with the methanogenesis/WLP-based divergences (Fig. 3). It has been suggested that LACA was (hyper)thermophilic while a number of independent evolution to mesophily occurred within archaea (44) and the methanogenic ancestor was also hyperthermophilic (12). Here, by predicting optimal growth temperature from extant archaeal genomes, we observed that some of the mesophilic development coincided with loss of methanogenesis. The archaeal lineages that vertically inherited methanogenesis (Euryarchaeota and TACK) contain deep-branching hyperthermophilic clades and internal mesophilic clades. In contrast, the ancestors that lost methanogenesis (Asgardarchaeota and DPANN) do not have hyperthermophilic clades at their basal positions, suggesting each ancestor might emerge in a cooler environment, contradicting with previous reports (45). In agreement with the prediction based on extant archaeal genomes, we constructed the ancestral 16S rRNA sequences and found that each ancestor of Euryarchaeota and TACK was hyperthermophilic (79 and 85°C, respectively) while those for Asgardarchaeota and DPANN had lower growth temperature (67 and 42°C respectively). Genome-based quantification of biosynthetic capacity and optimal growth rate further shows that extant members of DPANN and Asgardarchaeota generally have reduced biosynthetic capacities and slow growth compared with Euryarchaeota and TACK (Table S2).

The gradients of physiological features clearly point toward an association of inheritance/loss of methanogenesis with major lifestyle transitions. One plausible interpretation is that, while organotrophy likely experienced thermodynamic inhibition from the high concentrations of geogenic H_2_ (e.g. in hydrothermal vents), the migration of some lithoautotrophic methanogen lineages toward lower temperature environments away from the point source of geogenic H_2_ created new opportunities for organotrophy by providing primary production and H_2_ scavenging under lower ambient H_2_ concentrations (2), giving way to the emergence of Asgardarchaeota and DPANN. In parallel, the reduced biosynthetic capacities and slow growth in Asgardarchaeota and DPANN may have resulted from the dependent and symbiotic organotrophic lifestyle that was developed at their ancestors (i.e. the Black Queen Hypothesis (46)). We observed complete loss of WLP and a pronouncedly steep decrease in biosynthetic capacity at Heimdallarchaeia (boxed in Fig. 3), suggesting a critical transition to stronger catabolic and anabolic dependency on symbiotic partners. Considering it is the closest relative to Eukarya's archaeal ancestor reported to date (47), we suspect that the above lifestyle transition toward low energy, slow growth, lower temperature, and symbiosis may have contributed to initiation and, potentially, maturation of (endo)symbiosis with bacterial partners (48).

In summary, we clarified several key questions about the evolutionary history of methanogenesis. Methanogenesis began from primordial protein-free F_430_-driven reactions and, as an enzyme integrating F_430_ into its core emerged (LCA of MCRpf), multiple duplications/functional diversifications yielded proteins specific to particular alkane. The emergence of a methane-specific MCRpf complex (i.e. Mcr) coincided with LACA, a versatile methanogen capable of reducing CO_2_ and methylated compounds, suggesting the innovation of Mcr was a critical event in the evolution of *Archaea*. Modification of methanogenesis and the WLP, among many other potential evolutionary events that remain to be discovered, paralleled early archaeal diversification, whose impact is still visible in extant organisms, depicting potentially important components of the evolutionary path from primordial life toward extant archaea.

## Materials and methods

### Phylogenetic analysis

Archaeal genomes that are included in the GTDB Release 202 (27) were downloaded from NCBI. Preliminary annotation of the genome assemblies was performed using Prokka v1.14.5 (49). Archaeal Clusters of Orthologous Genes (arCOGs) were annotated using eggnog-mapper v2.1.6 (50) against the eggNOG 5.0 database (51) with default parameters. To reduce redundancy under each arCOG, sequences from organisms belonging to the same family (based on GTDB taxonomy) were clustered using CD-HIT v4.8.1 (52) with a 60% similarity cut-off. Clustered sequences were aligned using MAFFT v7.487 (53) with the “localpair” mode and trimmed using trimAl v1.4 (54) with a gap threshold of 0.8. Phylogenies of single proteins were inferred using the maximum-likelihood method and constructed using IQ-tree v2.2.0 (55). Model finding implemented in IQ-tree was performed to find the best-fit protein mixture model (C10–C60) according to Bayesian Information Criterion. The resulted ultrafast bootstrap (1,000 replicates) values were subject to bootstrap expectation transfer (tbe) using BOOSTER v0.1.2 (56). Phylogenies of concatenated proteins were subject to Bayesian inference using PhyloBayes MPI v1.9 (57) (-cat -gtr). A consensus tree is summarized from two independent chains that reached convergency after 5,000 iterations (maxdiff <0.2 under a burn-in of 1,000).

An archaeal species tree was constructed using a concatenated alignment of 30 universally conserved ribosomal proteins (40) from representative genomes and maximum-likelihood estimation (IQ-TREE v2.2.0) based on a universal distribution mixture (UDM) model with 64 components and LCLR transformation constructed with the HOGENOM and HSSP databases (-m Poisson + UDM0064LCLR) (58). The UDM model was chosen over the C60 mixture model often used in ribosomal protein tree calculation as it has been shown to have improved model fit and performance (58, 59). Representative genomes were selected for each archaeal family and bacterial phyla, only if the taxonomic group included species representative genomes in the GTDB r202 database that met one of the following criteria: (i) cultured organisms with ≥90% completeness, ≤5% contamination (as estimated by CheckM (60)), and ≤20 contigs; (ii) uncultured organisms with ≥85% completeness, ≤3% contamination, and ≤20 contigs; and (iii) Patescibacteria with ≥60% completeness and ≤1 contig. The same alignment was also used for Bayesian inference with PhyloBayes (-cat -gtr). Eight independent chains were calculated for 10,000 iterations, but no chain converged with another, and each chain exhibited different topologies.

Gene tree–species tree reconciliation was conducted for the three key phylogenies, i.e. CfbABCDE, MtrAH, and McrBDCGA, using Ranger-DTL v2.0 (61), which requires a rooted species tree (the maximum-likelihood species tree calculated above) and a rooted gene tree. There are multiple approaches to root a gene tree, such as the outgroup method, mid-point rooting method, minimal ancestral deviation method (MAD) (62), and reconciliation-based method, among which we prioritize the results based on outgroup sequences/homologs. When such outgroups were not available, we adopted the rooting scenario supported by at least two methods and the majority of subunits. For Mtr, tree rooting was performed through (i) outgroup-based rooting of the catalytic subunits MtrAH with functionally distinct homologs MtxAH and (ii) reconciliation-based rooting for all subunits (except for subunits F and G) using Treerecs (a consensus tree was calculated using IQ-TREE from 1,000 re-rooting scenarios on the last 1,000 Bayesian iterations generated by Treerecs v1.2) (63). For CfbABCDE, tree rooting was performed through (i) outgroup-based rooting of four of the five proteins (CfbA/B/D/E) and (ii) reconciliation-based rooting (via Treerecs) of a tree based on concatenated alignment of all five proteins. For McrBDCGA that lack outgroup sequences/homologs, tree rooting was performed through reconciliation- and MAD-based rooting and on three trees—all subunits, catalytic subunits McrBGA, and supportive subunits McrDC. The above analyses generated consistent rooting results. Despite the caution we took here, it should be noted that the rooting we found only represent an optimal hypothesis, while alternative scenarios may be possible (Supplementary Information). After we determined the rooting of each gene tree, we optimized the costs for duplication (*D*), transfer (*T*), and loss (*L*) that Ranger-DTL uses to explain the gene-tree topology given the species tree. We first determined the *T*:*L* ratio by running OptRoot under different *T* values, each of which generated multiple optimal rootings for the gene tree. We inspected the most parsimonious *T* value that allowed OptRoot to recover the rooting observed from the above analyses. Using this *T* value (*T* = 10 for CfbABCDE, *T* = 8 for MtrAH, *T* = 16 for McrBDCGA; *L* = 1 for all), we ran Ranger-DTL with varying *D* values (i.e. ¼ *T*, ½ *T*, ¾ *T*; 100 runs) and compiled the results using AggregateRanger into a single reconciliation result with support values for the inferred events, as recommended by Ranger-DTL's manual.

### Other calculations

Optimal growth temperature of extant archaea was predicted from genome assembly using Tome v1.0.0 with default parameters (64). In addition, to calculate the growth temperature of ancestral nodes, 16S rRNA gene sequence of all archaea were downloaded from the SILVA databased and was used to reconstruct ancestral sequence using the BppAncestor function in the BppSuite v2.4.1 package (65). The growth temperature of the ancestor of major lineages was estimated based on 16S rRNA uracil content using a previously reported correlation (66). Maximum growth rate (minimal doubling time) was predicted using the R package gRodon (67) based on genome assembly and the optimal growth temperature predicted above. We compared the predicted minimal doubling time with their doubling time reported in literatures and found that, in general, those predicted to be slow growers were also reported to grow slowly (Supplementary Information). De novo biosynthesis of 22 amino acids and 14 vitamins/cofactors (from basic building blocks such as those produced during glycolysis) was annotated manually based on the arCOG assignment results and pathway information in MetaCyc database (68). A genome is allowed to miss a small fraction of the pathway to be considered as possessing the synthesis capacity according to the following rule: 0 missing gene is allowed for pathways with ≤3 genes, 1 for 4–5 genes, 2 for 6–10 genes, 3 for 11–15 genes, 4 for 16 + genes. Quasi-equilibrium thermodynamic calculation was performed as described previously (Table S1) (69) using reported standard free energy change (ΔG^o^′) (70). To estimate the intermediate concentrations, we considered thermodynamic equilibrium (Δ*G* = 0) for most individual steps, but also energy conservation/dissipation (Δ*G* < 0) and energy investment (Δ*G* > 0) when necessary. Several assumptions and justifications were made during the calculation. Cofactors (methanofuran, methanopterin, coenzyme M, and coenzyme B) were presumed to remain a constant total concentration summarized from multiple references (71–74), while the concentrations of free or ligated forms varied. Iterations were considered to reach equilibrium when the sum of all forms of a cofactor matches the total concentration presumed above. The ratio between oxidized and reduced electron carriers was determined based on an equilibrium with the H_2_ partial pressure.

### Purification of cofactor F_430_ and autocatalytic test

To extract and purify cofactor F_430_, *Methanosarcina barkeri* type strain (DSM 800) was cultured in a 10-L fermenter. Wet cells were sonicated with 1% formic acid on ice and supernatant was recovered by centrifugation (15,000 × *g* for 30 min). The supernatant was passed though Q sepharose fast-flow column that was pre-equilibrated with 50 mM tris-HCl buffer. F_430_ in the solution was concentrated in a C18 SPE column pre-equilibrated with 1% formic acid and eluted with methanol. The F_430_ fraction was further purified via two stages of liquid chromatography using Agilent 1260 HPLC equipped with a diode array detector and a fraction collector as following. The first HPLC purification was performed with Chromolith Semi Prep RP-18e (10 mm × 100 mm; Merck). Eluents were 100 mM NaClO_4_/HClO_4_ (pH 2.3) for A and acetonitrile for B. The gradient condition was 0% B at 0 min, 20% B until 2 min, then 27.3% B until 30 min at 1 mL/min of flow rate. The collected F_430_ fraction was desalted using a C18 SPE column under the same condition described above. The second purification was performed with Hypercarb (10 mm × 150 mm; Thermo Scientific). Eluents were HClO_4_aq (pH 1) for A and acetonitrile for B. The gradient condition was 0% B at 0 min, 30% B until 3 min, then 52.8% B until 70 min at 1 mL/min of flow rate. The collected F_430_ fraction was desalted using an Oasis HLB SPE column.

One μmol of purified F_430_, 0.2 µmol of methyl- and benzyl-viologen, 100 μmol of Ti(III)-citrate and methyl substrate were dissolved in previously degassed CAPS buffer solution (totally 10 mL) in a 30 mL glass vial and sealed with a butyl rubber stopper and an aluminum cap. The vial was standing at room temperature. Head space gas was periodically sampled for methane measurement using an HP 6890 GC equipped with a flame ionization detector. The compounds were separated with a PoraBOND Q column (25 m, 0.53 mm i.d., 10 μm film thickness; Agilent) using helium as the carrier gas at a flow rate of 8.5 mL/min. Initial oven temperature was 35°C and was heated to 190°C by 20°C/min. Injection was performed in split mode (split ratio 5:1). Concentration was determined by a C_1_–C_4_ alkane gas standard.

## Supplementary Material

pgad023_Supplementary_Data

## Data Availability

Genomes analyzed in this manuscript are available in GTDB and NCBI. The accession numbers are provided in supplementary data. For or all phylogenetic trees, the original and trimmed alignments and the corresponding trees in Newick format are available at https://github.com/meiranmeiran/archaeaDataset.
