# Supplementary material for: The origin and evolution of methanogenesis and Archaea are intertwined
Source: PNAS Nexus. 2023 Jan 31;2(2):pgad023. doi: 10.1093/pnasnexus/pgad023 (PMC9982363; doi:10.1093/pnasnexus/pgad023)
Supplement: pgad023_Supplementary_Data [file pgad023_supplementary_data.zip › PNASNEXUS-PNASNEXUS-2022-01050-s01.pdf]

Supplementary Information for

The origin and evolution of methanogenesis and Archaea are intertwined

Ran Mei, Masanori Kaneko, Hiroyuki Imachi, and Masaru K. Nobu*

* Masaru K. Nobu

Email: m.nobu@aist.go.jp.

This PDF file includes:

Supplementary text

Figures S1 to S32; Tables S1 to S2; Legends for Datasets S1; SI References.

Other supplementary materials for this manuscript include the following:

Datasets S1

Supplementary Text

An evolving view of the evolutionary history of methanogenesis

The notion of methanogenesis being an ancient metabolism is not new. More than half century ago, Thauer and colleagues predicted that "methane-forming bacteria and the clostridia are closest to the primordial anaerobes" based on physiological features such as energy production and tetra-pyrrole synthesis (1). Molecular evolutionary studies of methanogenesis also started in the 1970s, when Woese and colleagues in their prophetic work analysed a handful of ribosomal RNA sequences and proposed that the divergence between *Archaea* and *Bacteria* was the most ancient phylogenetic event (2, 3). These authors envisioned that methanogenesis "was peculiarly well suited to the projected primitive atmosphere of this planet" (4).

While the antiquity of methanogenesis is seldomly challenged, understanding of its evolutionary history is constantly evolving (5). Given methanogens were only found within Euryarchaeota for decades, an euryarchaeotal origin of methanogenesis was commonly accepted (6), which was further substantiated by the phylogenetic placement of *Methanopyrus kandleri* (7, 8) and Methanomassillicoccus (9) that are basal in Euryarchaeota. This view was altered by the discoveries of several non-euryarchaeotal methanogens in the TACK branch of the archaeal domain. Methanomethyliaceae was first found to possess the Mcr complex and form a robust monophyly in Mcr phylogenetic tree, suggesting that methanogenesis antedated the origin of Euryarchaeota (10). Another two TACK lineages were later predicted with similar physiological traits and phylogenetic placement, i.e., Methanodesulfokores (11) and Methylarchaeum (12). The discovery of these TACK methanogens consolidates an ancient origin of methanogenesis, no later than the divergence of Euryarchaeota and TACK (12-16), which is in line with the long-standing biochemical inferences of methanogenesis as a primitive metabolism (1, 4, 17-20). **However, the precise chronology of methanogenesis is not yet conclusive.** Was the first methanogen the last common ancestor of Euryarchaeota and TACK, or an earlier archaeon such as the last common ancestor of all archaea? Was biological methanogenesis present earlier than the first methanogen?

The discoveries of TACK methanogens elicit another debate on the original form of methanogenesis. Among several variances of methanogenesis, the one that reduces CO_2 via the Wood-Ljungdahl pathway (WLP) predominates the phylogenetic distribution of methanogens. However, TACK methanogens are deep-branching in both genome and Mcr gene tree and most of them likely reduce methylated compounds instead of CO_2, suggestive of a methylotrophic origin of methanogenesis (14). Other evidence supporting this argument includes (i) it is a simpler

pathway compared to the CO_2-reducing form that additionally requires the WLP and the Mtr complex; (ii) Mcr sequences of methylotrophic Euryarchaeota are also deeper branching than those of canonical CO_2-reducing methanogens; (iii) Mtr seems a late innovation within Euryarchaeota; (iv) methyl-reducing methanogenesis is compatible with the ancient earth that might be rich in methylated compound. Nonetheless, a contradicting scenario, i.e., CO_2-reducing methanogenesis being the origin, was proposed based on the discovery of the CO_2-reducing Methanohydrogenales in TACK and its firm monophyly in the Mtr phylogeny (15). Furthermore, the rooting of the archaeal species tree between CO_2-reducing methanogens (21, 22) and the antiquity of the WLP (23, 24) are both indicative of CO_2-reducing methanogenesis being the ancestral type (13). **Apparently, there is yet no consensus on which form represents the ancient origin, and which represents a later adaptation.**

Another unsettled debate is the relationship between Mcr and its diverging homologous that are involved in activating non-methane alkanes (Acr), which was first found in Bathyarchaeota (25) and later in diverse archaea (13, 26-33). Various interpretations of the detailed evolutionary path of this expanding protein family have been made. Borrel et al. argued that the divergent Mcr homologs evolved due to change in function and adaptation to non-methane substrates, i.e., later evolution arising from canonical Mcr (13). Similarly, Hua et al. suggested that Acr emerged from a duplicated Mcr with neofunctionalization that occurred within Euryarchaeota, because there are more euryarchaeotal sequences in the divergent cluster (12). However, it was also proposed that Mcr and Acr could possibly originate from an ancient divergence prior to the divergence of TACK and Euryarchaeota (14). The same authors also proposed a number of alternative scenarios where Acr and Ecr evolved from within the Mcr clade, Acr evolved from an ancient methyl-binding enzyme at the root of the protein family (14), independent horizontal transfers in early evolution, or convergent evolution from canonical mcr under positive natural selection (28). **Therefore, the ancestral physiology of the broad alkyl-CoM reductase protein family remains elusive.**

Last but not the least, while it is generally accepted that methanogenesis has gone through complex inheritance, modification, and loss (16, 34), **how is the evolution intertwined with the diversification of the Archaea domain is not yet explicitly demonstrated.** The enriched understanding of methanogenesis itself and expanding archaeal tree, including both methanogenetic and non-methanogenic lineages, now provide a good opportunity to investigate the two in parallel and unravel key evolutionary process of the domain.

Uncertainty about the deep divergence in the archaeal genome tree

Constructing the correct archaeal genome tree is critical but remains challenging. For example, the placement of several archaeal lineages are still contested, including Nanohaloarcheaota (35, 36), Altiarchaea (37, 38), and Methanonatronarchaeia (39-42). One of the most prominent challenges of constructing the archaeal genome tree is to resolve the deepest divergence of the domain, i.e., where the root should be placed (34, 43).

At least three ways of rooting have been proposed. In rooting scenario A, Euryarchaeota and the TACK superphylum are the deepest divergence in *Archaea* (44-46). In scenario B, the most common rooting reported in recent years, the rooting is between DPANN and all other archaea (36, 47-50). However, the deep placement DPANN is often confronted by its reduced genome, elevated rate of sequence evolution (43), and the parasitic lifestyle related phylogenetic artifact (51). Rooting scenario C was proposed using a divide-and-conquer approach (21, 22, 34), which firmly recovered the placement of important novel archaeal lineages and Eukarya but precluded the monopoly of Euryarchaeota. Consequently, the first divergence of Archaea was placed between Cluster I (TACK, Asgardarchaeota, Thermococci, Methanobacteriota, Altiarchaea) and Cluster II (Halobacteriota and Thermoplasmatota).

In line with the ongoing debate, our phylogenomic analysis reveals uncertainty in the root of the archaeal domain. Although DPANN is placed at the basal place of the domain based on maximum-likelihood inference, the supports are low. Eight independent chains of Bayesian inference of the same alignment could not converge but generate various deep topologies. The inconsistence between Maximum-likelihood and Bayesian inference on the same super matrix is also recently observed (22). It is plausible that Euryarchaeota, DPANN, and TACK/Asgardarchaeota might diverged, if not simultaneously, in a short period of time.

However, the uncertainty of the root placement does not preclude the inference of the origination of most proteins discussed in our study. Taking the phylogeny of CfbABCDE as an example, a robust Euryarchaeota-TACK divergence is observed with verticality in both sides of the divergence. Under rooting scenario A where Euryarchaeota is the first divergence, CfbABCDE is vertically present in both sides of the genome tree and, thus, it is present prior to the divergence, i.e., at LACA. Similarly, under rooting scenario C, CfbABCDE is present in both sides of the genome tree (Cluster I and Cluster II), and, thus, it is present at LACA. Under rooting scenario B where DPANN is the first divergence, CfbABCDE could be either present at LACA and lost by DPANN or was invented after DPANN diverged. We believe the latter is less likely because CfbABCDE, as well as many other functions related to methanogenesis, would have to be

invented in the short period of time after DPANN diverged and before the ancestor of TACK-Euryarchaeota emerged. It is also reasonable to speculate that LACA possessed CfbABCDE and DPANN lost it, considering that DPANN has gone through substantial genome reduction and functional loss. Overall, it is logic to conclude that methanogenesis-related functions with a robust Euryarchaeota-TACK divergence (e.g., CfbABCDE, Mtr, Mcr, Mta) were present at LACA, regardless of the currently unsolved root of the archaea genome tree.

Confirmation of genome-based prediction of minimal doubling time

The growth behavior, e.g., minimal doubling time, of microorganisms is subject to many factors and is extremely difficult, if not impossible, to measure. For *Archaea*, the lack of cultured representatives for many lineages further stymie domain-scale comparison. We predicted the minimal doubling time based on all archaeal genomes using gRogon (52). To validate the prediction, we searched for the (optimal) doubling time of various archaea in literatures and compared their rank in reported doubling time and their rank in predicted doubling time. We observed a moderately positive correlation (R=0.59), i.e., those predicted to be slow growers are also reported to grow slowly. In addition, we also searched in the BacDive databased that is associated with DSMZ, where we found 39 species (not overlapping with species above) with both genome accession and incubation time, which is equivalent to doubling time. The incubation time is recorded as a range, e.g., 3-7 days, which precludes correlation test against our numerical prediction, so we classified the growth to fast (<7 days incubation), medium (7-14 days), slow (> 14 days). The average predicted doubling time for each category was respectively 4.17, 4.71, and 6.03 hours, indicating that predictions by gRodon can be used to compare growth rates.

Uncertainty related to reconciliation analysis

In order to predict key evolutionary events, including speciation, horizontal transfer, and duplication, that took place during the evolution of methanogenesis, we performed reconciliation-based analysis on key phylogenies, i.e., CfbABCDE, MtrAH, and McrBDCGA. While we have taken extreme caution in performing the analysis (as we described in the Method section in the main text), uncertainty and caveats related this analysis should not be underestimated. First, each gene tree has to be mapped to a rooted archaeal species tree, which is still not stable in several key positions as we have mentioned above. Second, the rooting of gene tree has decisive effects on the reconciliation results. Thus, we try to root each gene tree with multiple methods (e.g., outgroup, Treerecs, MAD, OptRoot, individual subunits, etc.) and seek for the most reasonable consensus.

However, there is always room for reevaluating the rooting, especially when more thorough sampling becomes available for clades poorly represented (or not sampled at all today), such as the TACK clade in the phylogenies of CfbABCDE and MtrAH, as well as the Ecr and Acr clades in the phylogeny of MCRpf. Last but not the least, key parameters such as the DTL costs we used are estimated based on currently observed topology of species and gene trees, which may change when novel lineages are discovered at critical phylogenetic positions.

Supplementary Figures

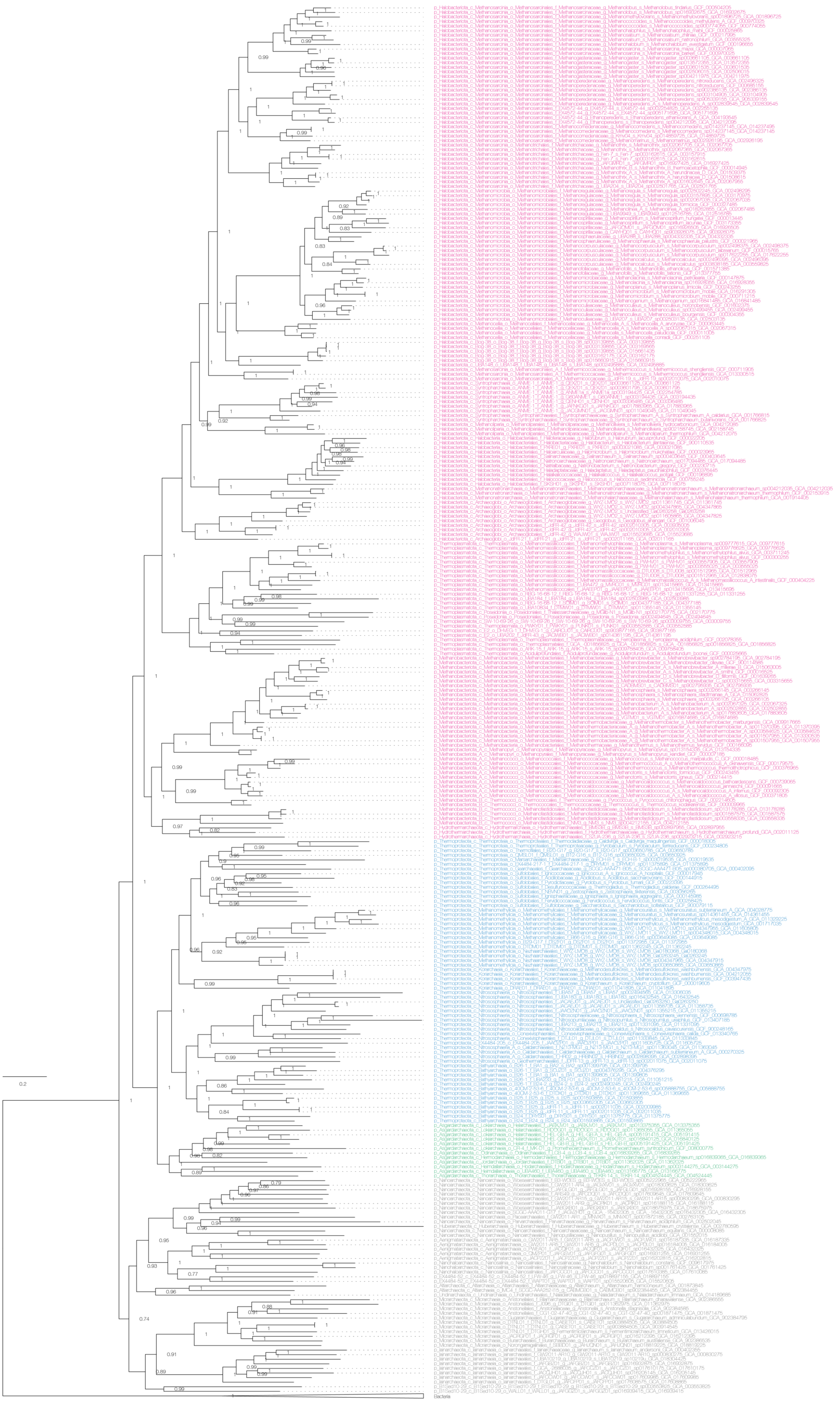

Figure S1. Archaeal phylogeny (uncollapsed version of Figure 1A). Maximum-likelihood inference was performed using IQ-tree (Poisson+UDM0064LCLR, 1000 ultrafast bootstrap replicates) on 30 conserved ribosomal proteins. Values on nodes are tbe-transformed ultrafast bootstrap values. Taxa are colored according to Euryarchaeota (pink), TACK (blue), Asgardarchaeota (green), and DPANN (grey). Bacteria are used as the outgroup. The datasets used to generate this tree (original and trimmed alignment) and the corresponding tree in Newick format are provided in Supplementary Datasets.

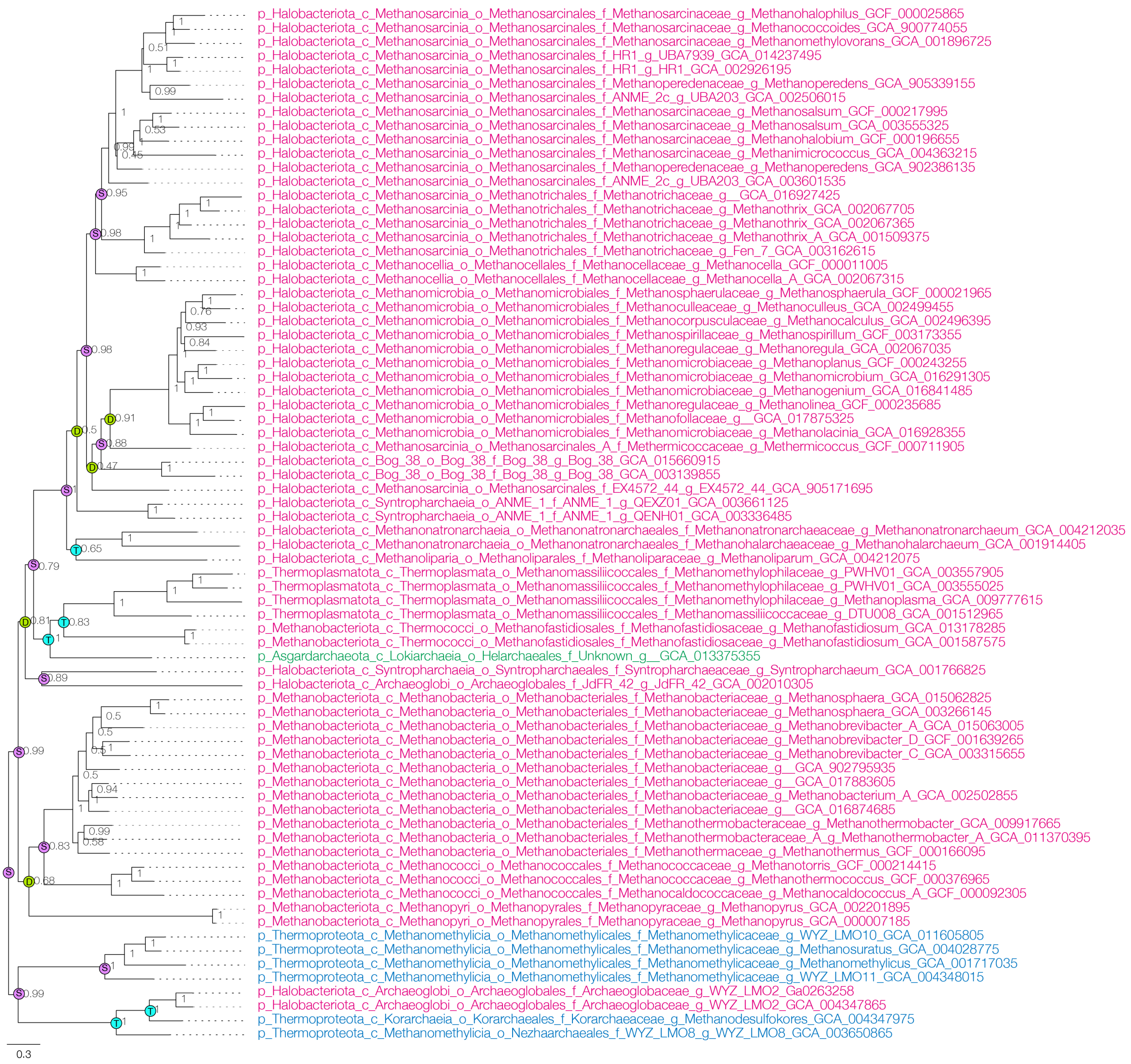

Figure S2. Phylogeny of CfbABCDE (uncollapsed version of Figure 1B). Bayesian inference was performed using PhyloBayes (-cat -gtr). The displayed tree is a consensus tree of two independent chains that converged after 5000 runs with a burn-in of 1000 (maxdiff<0.2). Values one nodes are posterior probabilities. Taxa are colored according to Euryarchaeota (pink), TACK (blue), and Asgardarchaeota (green). Reconciling this tree on a species tree reveals that TACK is placed as a basal divergence from Euryarchaeota (i.e., as the root of the tree), suggesting a history traced back prior to the TACK-Euryarchaeota divergence. Duplication (D), transfer (T), and speciation (S) events are denoted on deep nodes. The datasets used to generate this tree (original and trimmed alignment) and the corresponding tree in Newick format are provided in Supplementary Datasets.

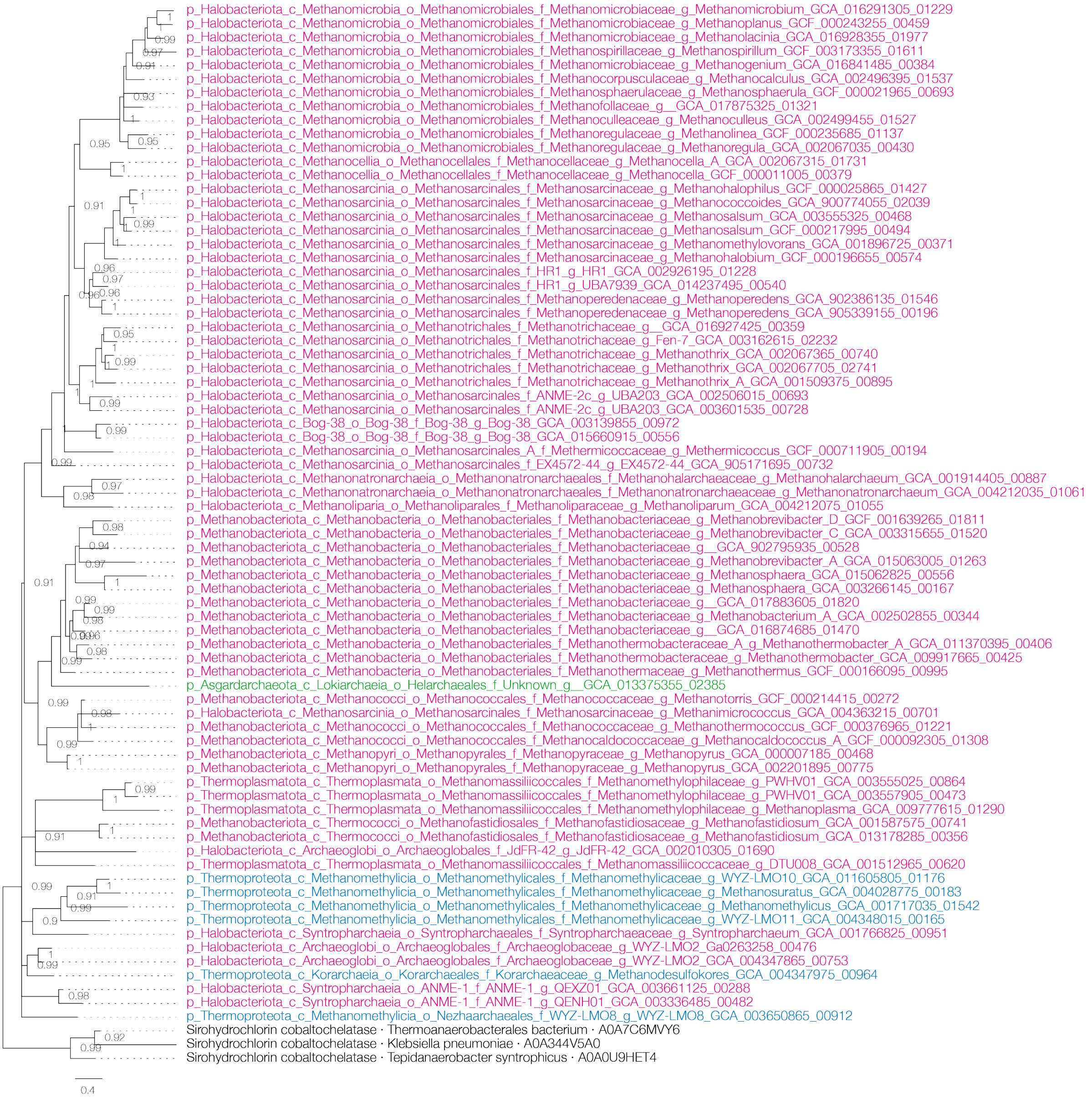

Figure S3. Phylogeny of CfbA for sirohydrochlorin cobaltochelatase. Maximum-likelihood tree is constructed using IQ-tree with LG+C10+G+F that is chosen as the best-fit model according to BIC. Values at nodes are tbe-transformed ultrafast bootstrap values. Nodes with <90% support are collapsed. Taxa are colored according to Euryarchaeota (pink), TACK (blue), and Asgardarchaeota (green). Bacterial sequences for sirohydrochlorin cobaltochelatase are used as the outgroup. Despite polytomy, TACK sequences are separated from the majority of Euryarchaeota. The datasets used to generate this tree (original and trimmed alignment) and the corresponding tree in Newick format are provided in Supplementary Datasets.

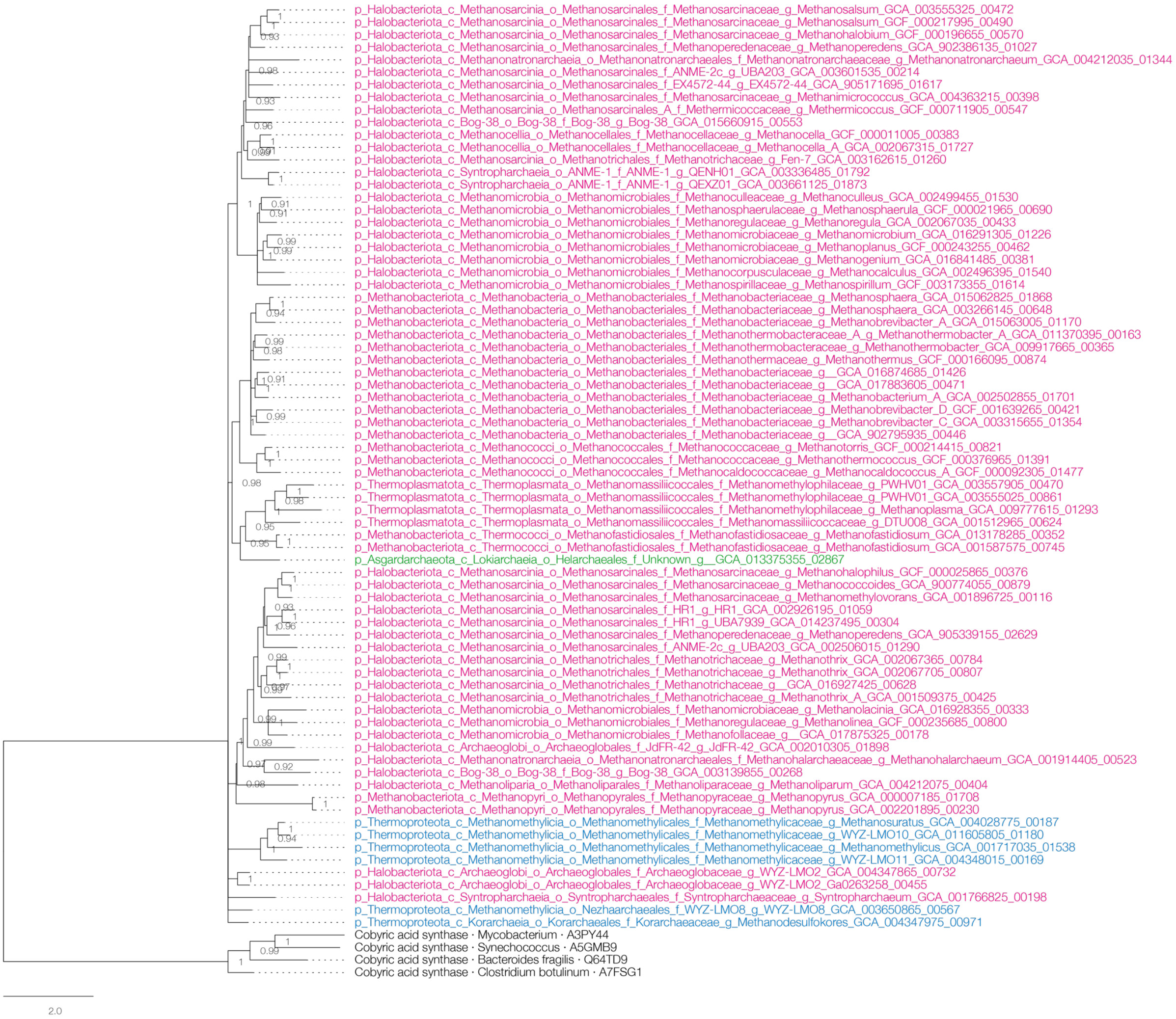

Figure S4. Phylogeny of CfbB for cobyrinate a,c-diamide synthase. Maximum-likelihood tree is constructed using IQ-tree with LG+C50+G+F that is chosen as the best-fit model according to BIC. Values on nodes are tbe-transformed ultrafast bootstrap values. Nodes with <90% support are collapsed. Taxa are colored according to Euryarchaeota (pink), TACK (blue), and Asgardarchaeota (green). Bacterial sequences for cobric acid synthase are used as the outgroup. Despite polytomy, TACK sequences are separated from the majority of Euryarchaeota. The datasets used to generate this tree (original and trimmed alignment) and the corresponding tree in Newick format are provided in Supplementary Datasets.

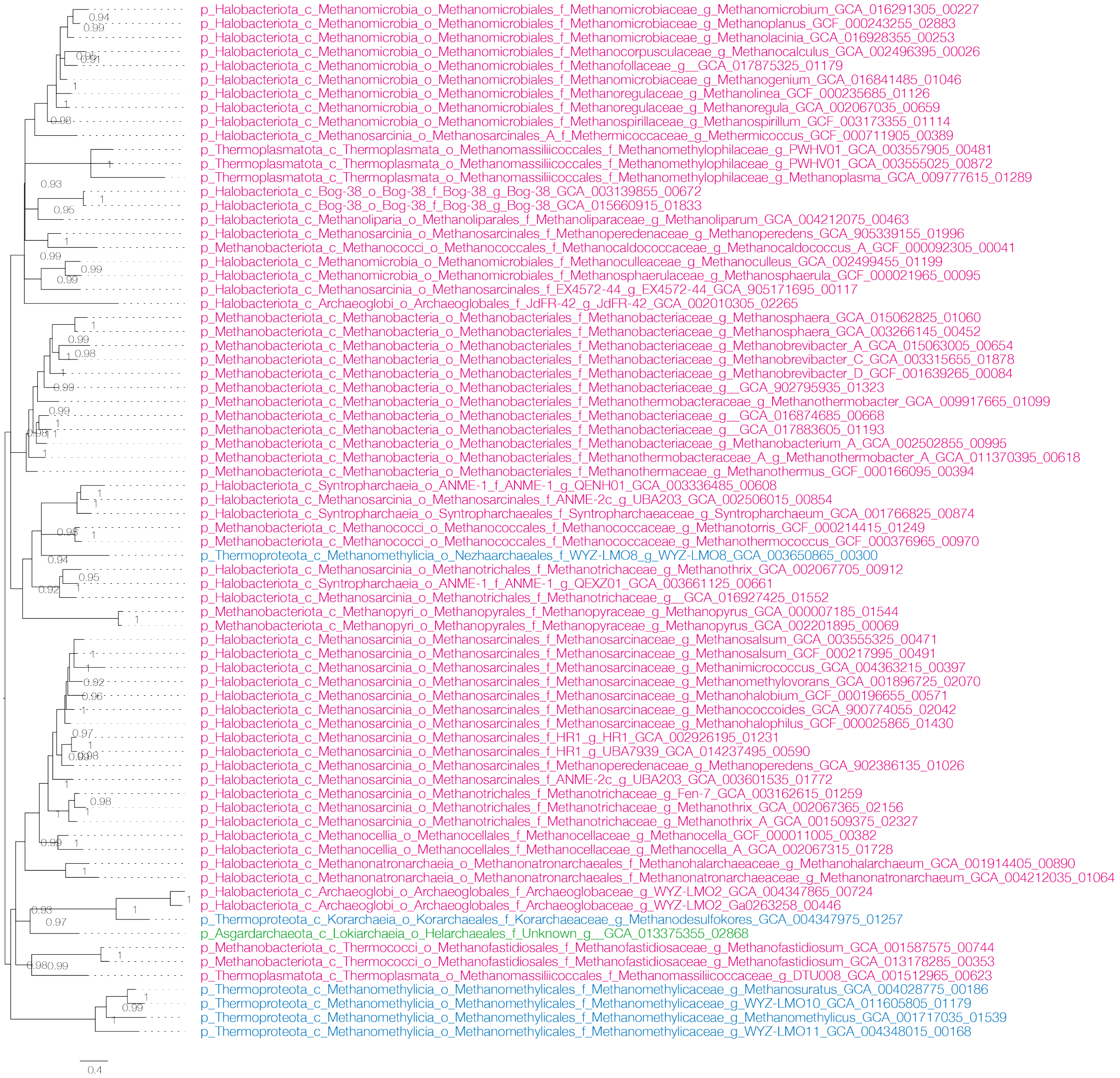

Figure S5. Phylogeny of CfbC for Ni-sirohydrochlorin a,c-diamide reductive cyclase complex. Maximum-likelihood tree is constructed using IQ-tree with LG+C20+G+F that is chosen as the best-fit model according to BIC. Values on nodes are tbe-transformed ultrafast bootstrap values. Nodes with <90% support are collapsed. Taxa are colored according to Euryarchaeota (pink), TACK (blue), and Asgardarchaeota (green). We were not able to find a bacterial homolog that could properly root the tree and the current tree is rooted based on midpoint rooting. The datasets used to generate this tree (original and trimmed alignment) and the corresponding tree in Newick format are provided in Supplementary Datasets.

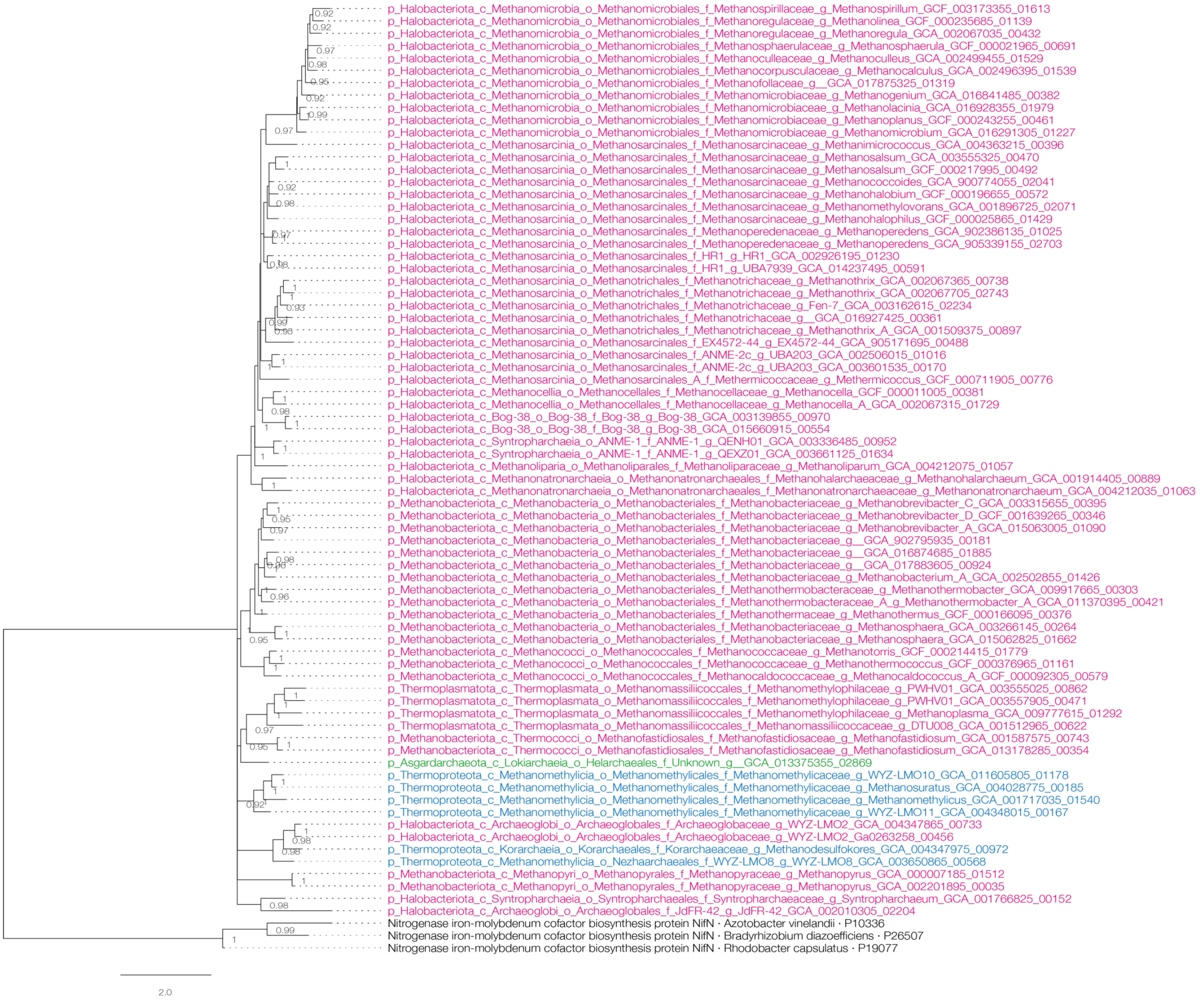

Figure S6. Phylogeny of CfbD for the Ni-sirohydrochlorin a,c-diamide reductive cyclase. Maximum-likelihood tree is constructed using IQ-tree with LG+C40+G+F that is chosen as the best-fit model according to BIC. Values on nodes are tbe-transformed ultrafast bootstrap values. Nodes with <90% support are collapsed. Taxa are colored according to Euryarchaeota (pink), TACK (blue), and Asgardarchaeota (green). Bacterial sequences for nitrogenase iron-molybdenum cofactor synthesis protein are used as the outgroup. Despite polytomy, TACK sequences are separated from the majority of Euryarchaeota. The datasets used to generate this tree (original and trimmed alignment) and the corresponding tree in Newick format are provided in Supplementary Datasets.

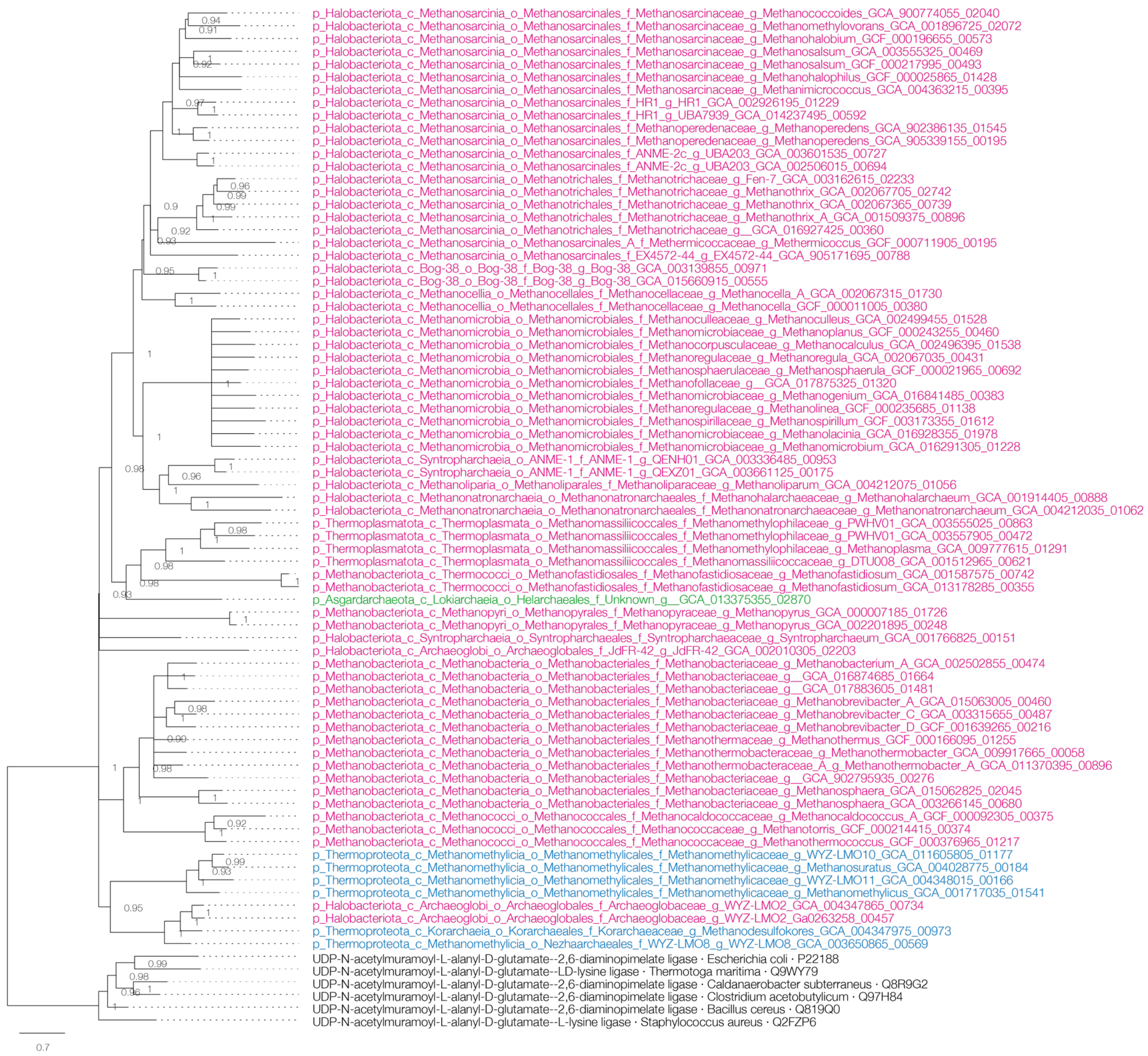

Figure S7. Phylogeny of CfbE for coenzyme F$_{430}$ synthetase. Maximum-likelihood tree is constructed using IQ-tree with LG+C40+G+F that is chosen as the best-fit model according to BIC. Values on nodes are tbe-transformed ultrafast bootstrap values. Nodes with <90% support are collapsed. Taxa are colored according to Euryarchaeota (pink), TACK (blue), and Asgardarchaeota (green). Bacterial sequences for UDP-N-acetylmuramoyl-L-alanyl-D-glutamate--2,6-diaminopimelate/lysine ligase are used as the outgroup. Despite polytomy, TACK sequences are separated from the majority of Euryarchaeota. The datasets used to generate this tree (original and trimmed alignment) and the corresponding tree in Newick format are provided in Supplementary Datasets.

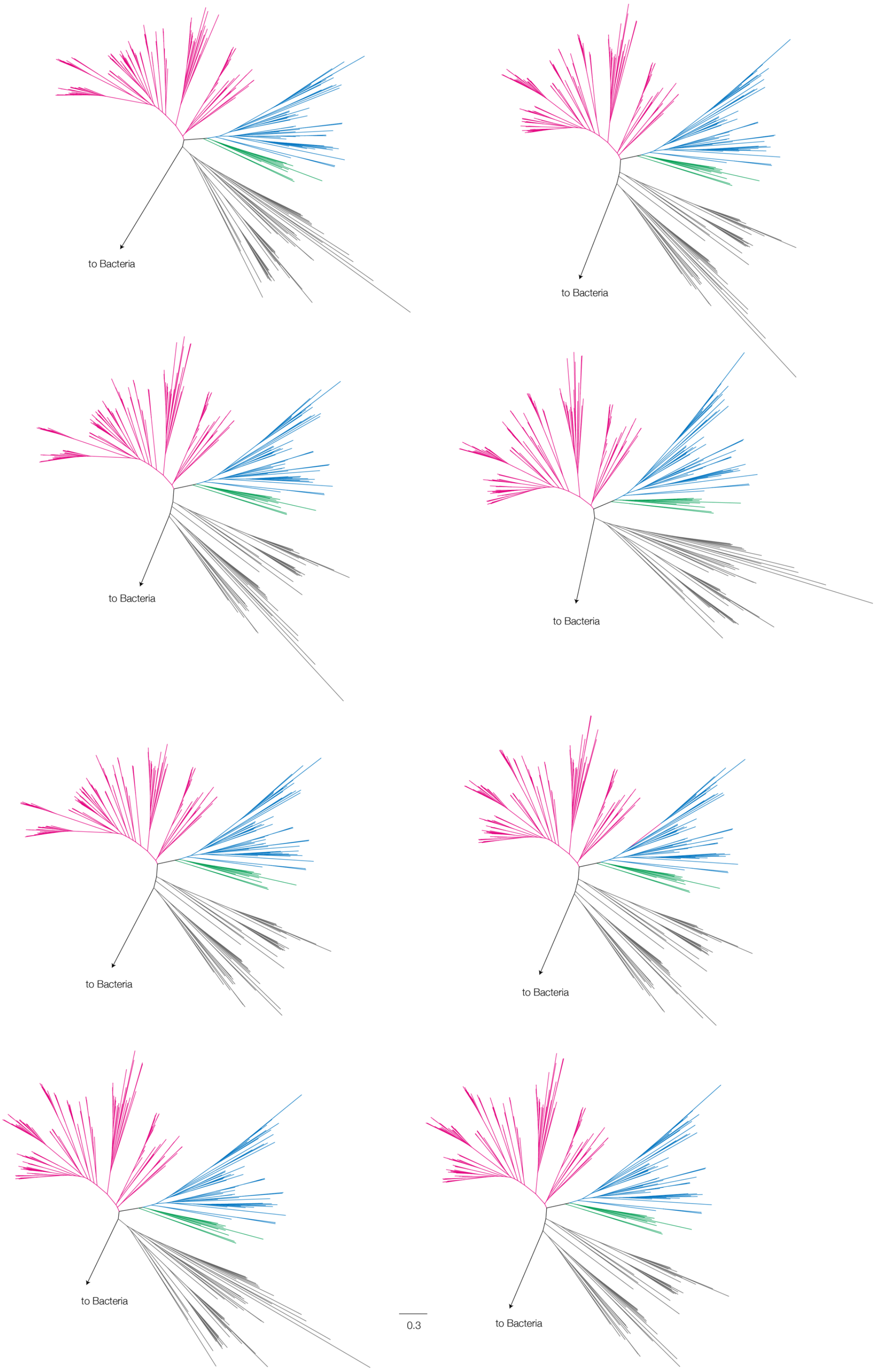

Figure S8. Bayesian inference of archaeal phylogeny. Bayesian inference of the same alignment used in Figure 1A and Figure S1 was performed using PhyloBayes (-cat -gtr). Eight independent chains were calculated but no chain could converge with another after 10,000 runs. Each revealed distinct topology (for example whether DPANN is recovered as monophyly). Clades are colored according to Euryarchaeota (pink), TACK (blue), DPANN (grey), and Asgardarchaeota (green). Bacteria is used as the outgroup. The datasets used to generate this tree (original and trimmed alignment) and the corresponding tree in Newick format are provided in Supplementary Datasets.

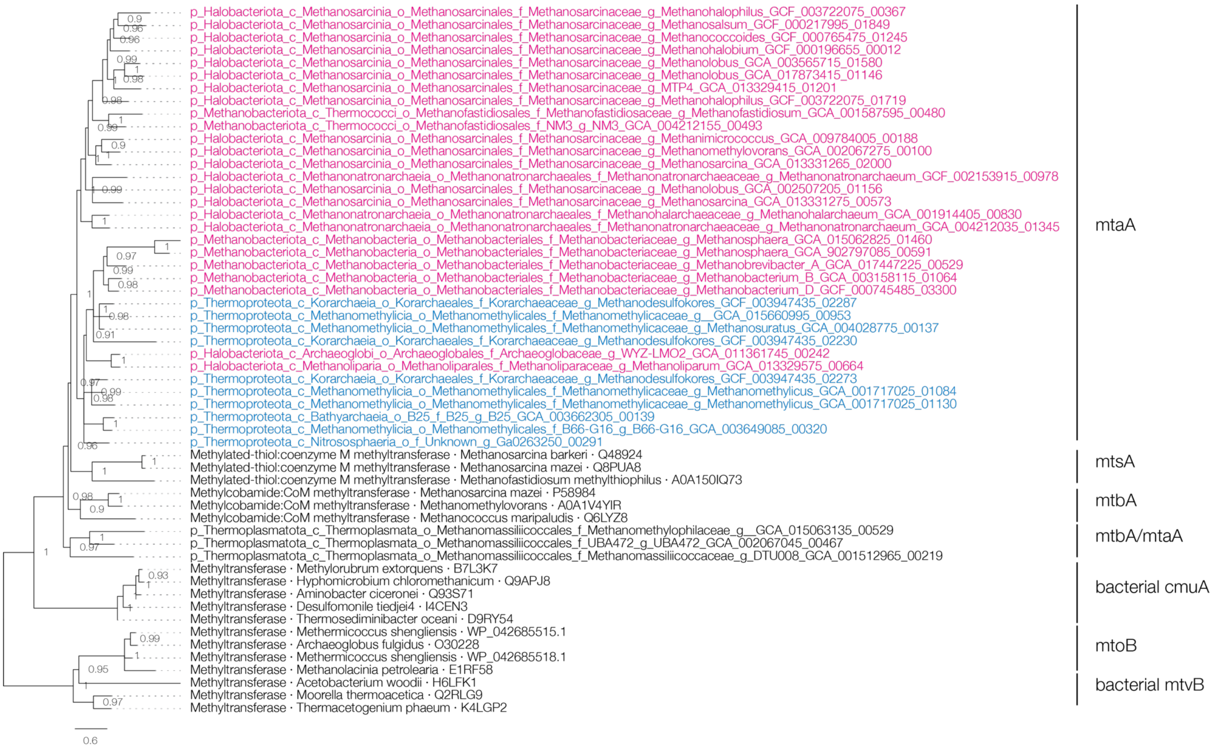

Figure S9. Phylogeny of MtaA for methylcobamide:CoM methyltransferase. Maximum-likelihood tree is constructed using IQ-tree with LG+C20+G+F that is chosen as the best-fit model according to BIC. Values at nodes are tbe-transformed ultrafast bootstrap values. Nodes with <90% support are collapsed. Taxa are colored according to Euryarchaeota (pink) and TACK (blue). Various methyltransferases are included and used as outgroup. Under this outgroup, TACK sequences (as well as some horizontally transferred Euryarchaeota) are placed as early divergence from most Euryarchaeota. The datasets used to generate this tree (original and trimmed alignment) and the corresponding tree in Newick format are provided in Supplementary Datasets.

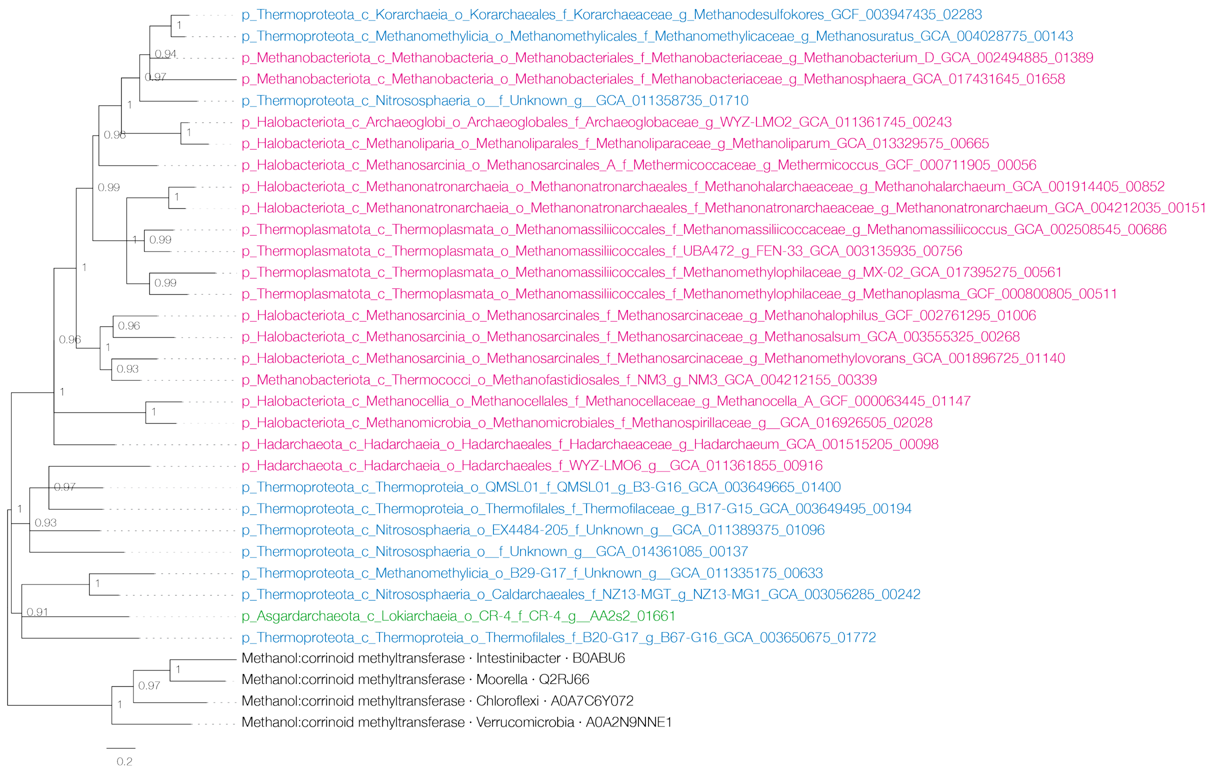

Figure S10. Phylogeny of MtaB for methanol:corrinoid methyltransferase. Maximum-likelihood tree is constructed using IQ-tree with LG+C30+G+F that is chosen as the best-fit model according to BIC. Values at nodes are tbe-transformed ultrafast bootstrap values. Nodes with <90% support are collapsed. Taxa are colored according to Euryarchaeota (pink), TACK (blue), and Asgardarchaeota (green). Bacterial methanol:corrinoid methyltransferase is used as outgroup. Under this outgroup, TACK sequences (as well as some horizontally transferred Halobacteriota and Asgardarchaeota) are placed as early divergence from most Euryarchaeota. The datasets used to generate this tree (original and trimmed alignment) and the corresponding tree in Newick format are provided in Supplementary Datasets.

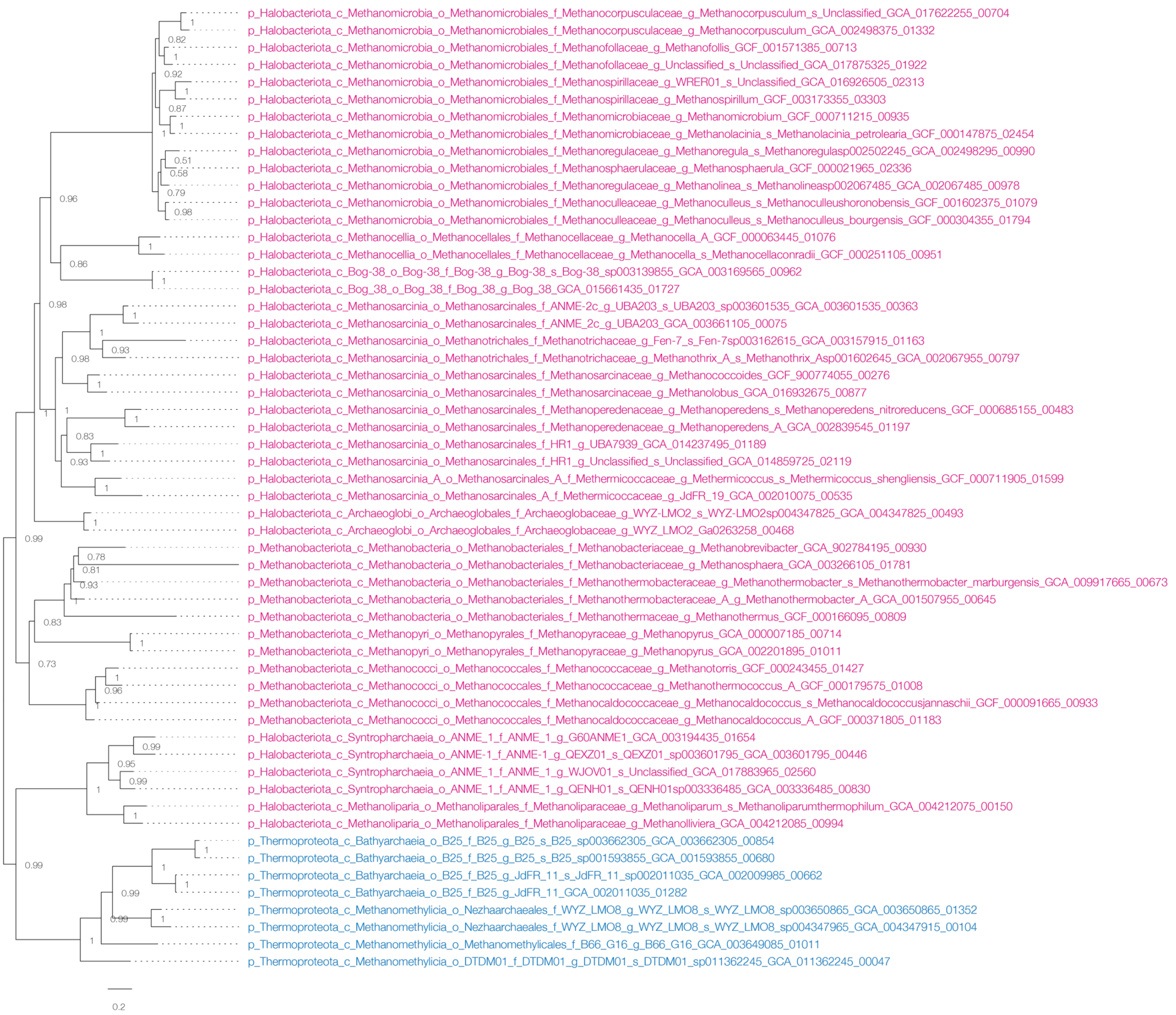

Figure S11. Phylogeny of MtrE for tetrahydromethanopterin S-methyltransferase subunit E. Maximum-likelihood tree is constructed using IQ-tree with LG+C30+G+F that is chosen as the best-fit model according to BIC. Values on nodes are tbe-transformed ultrafast bootstrap values. Taxa are colored according to Euryarchaeota (pink) and TACK (blue). TACK sequences (as well as some horizontally transferred Halobacteriota) are placed as basal divergence from most Euryarchaeota, supported by gene-species reconciliation. The datasets used to generate this tree (original and trimmed alignment) and the corresponding tree in Newick format are provided in Supplementary Datasets.

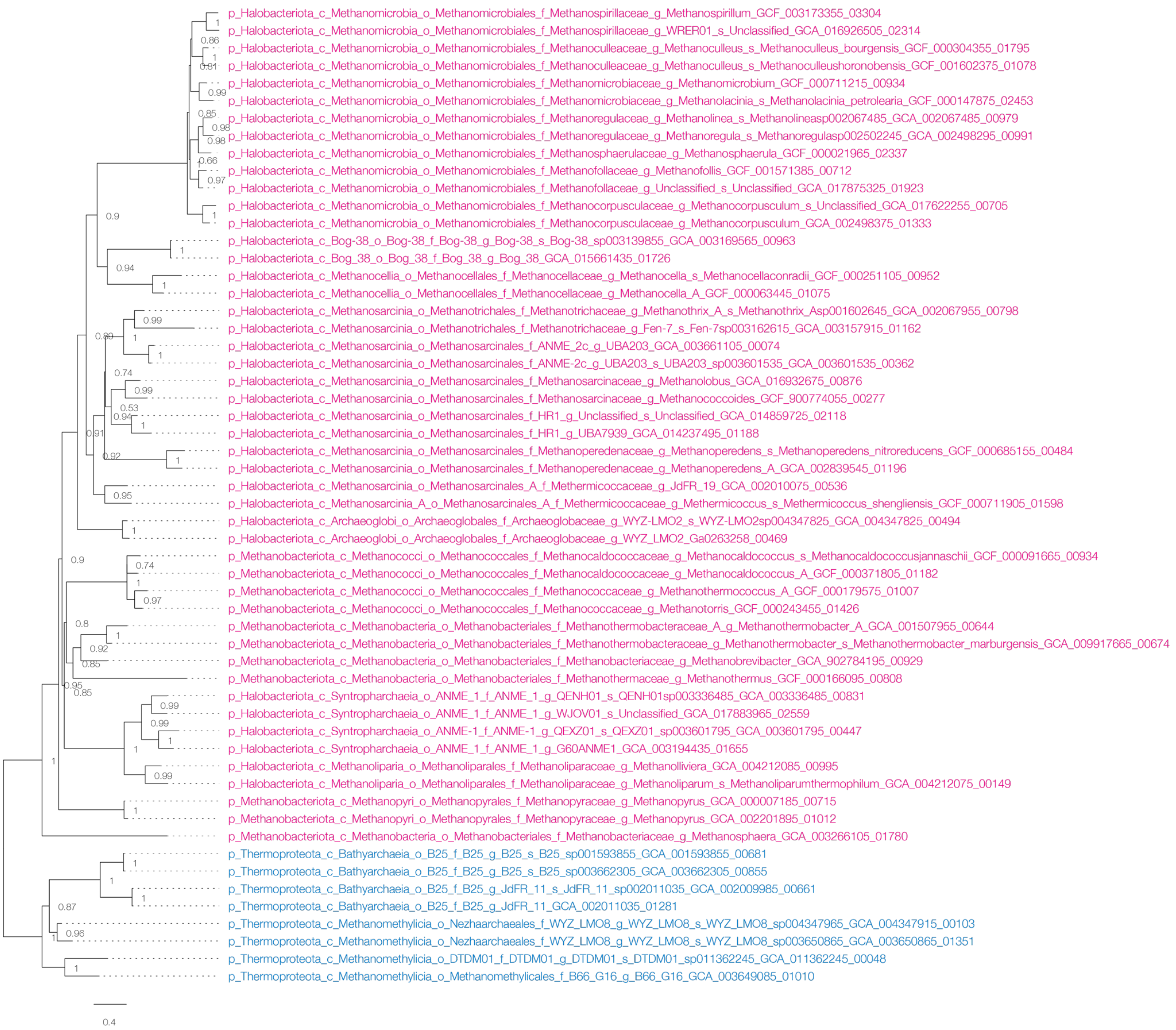

Figure S12. Phylogeny of MtrD for tetrahydromethanopterin S-methyltransferase subunit D. Maximum-likelihood tree is constructed using IQ-tree with LG+C20+G+F that is chosen as the best-fit model according to BIC. Values on nodes are tbe-transformed ultrafast bootstrap values. Taxa are colored according to Euryarchaeota (pink) and TACK (blue). TACK sequences are placed as basal divergence from Euryarchaeota, supported by gene-species reconciliation. The datasets used to generate this tree (original and trimmed alignment) and the corresponding tree in Newick format are provided in Supplementary Datasets.

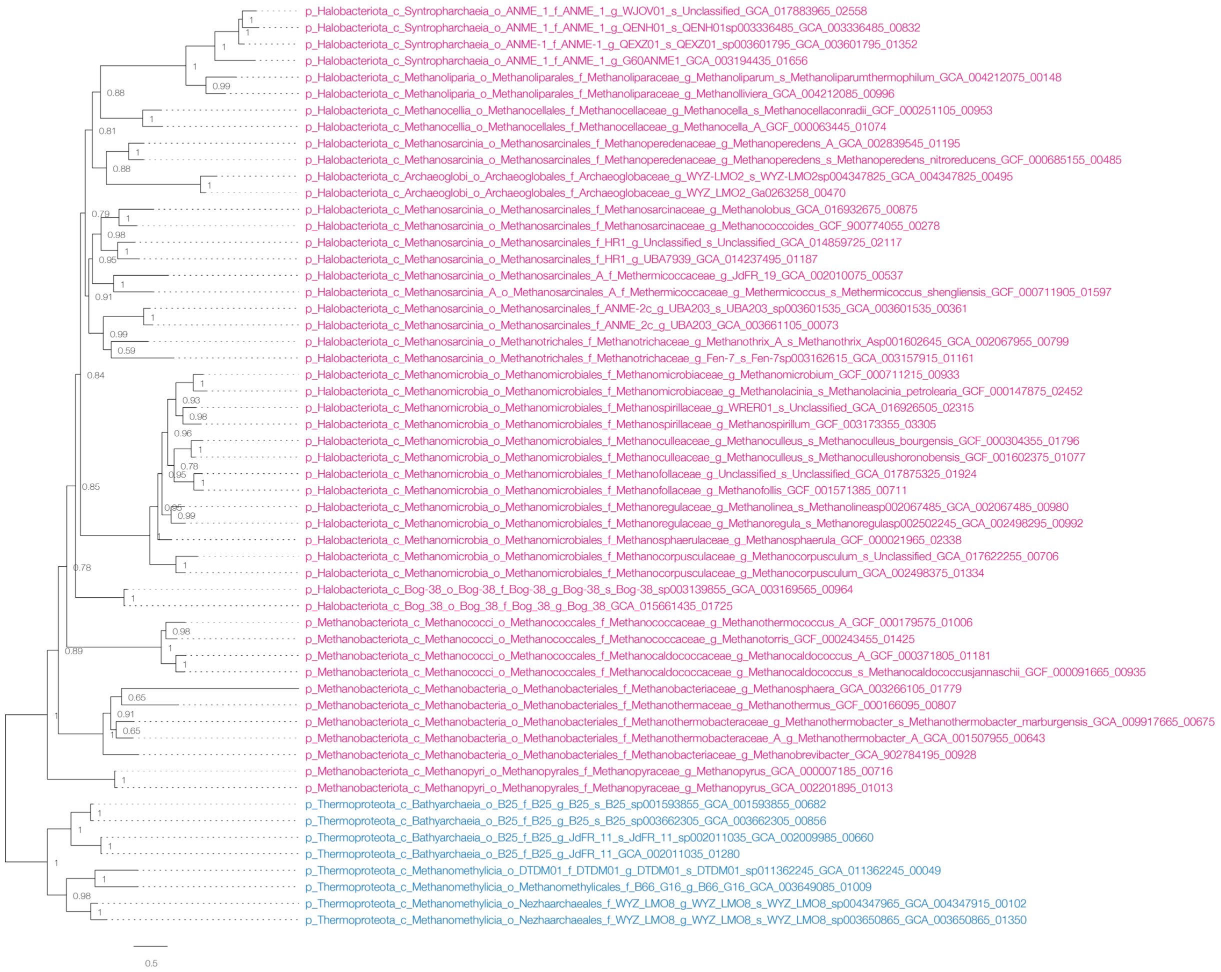

Figure S13. Phylogeny of MtrC for tetrahydromethanopterin S-methyltransferase subunit C. Maximum-likelihood tree is constructed using IQ-tree with LG+C30+G+F that is chosen as the best-fit model according to BIC. Values on nodes are tbe-transformed ultrafast bootstrap values. Taxa are colored according to Euryarchaeota (pink) and TACK (blue). TACK sequences are placed as basal divergence from Euryarchaeota, supported by gene-species reconciliation. The datasets used to generate this tree (original and trimmed alignment) and the corresponding tree in Newick format are provided in Supplementary Datasets.

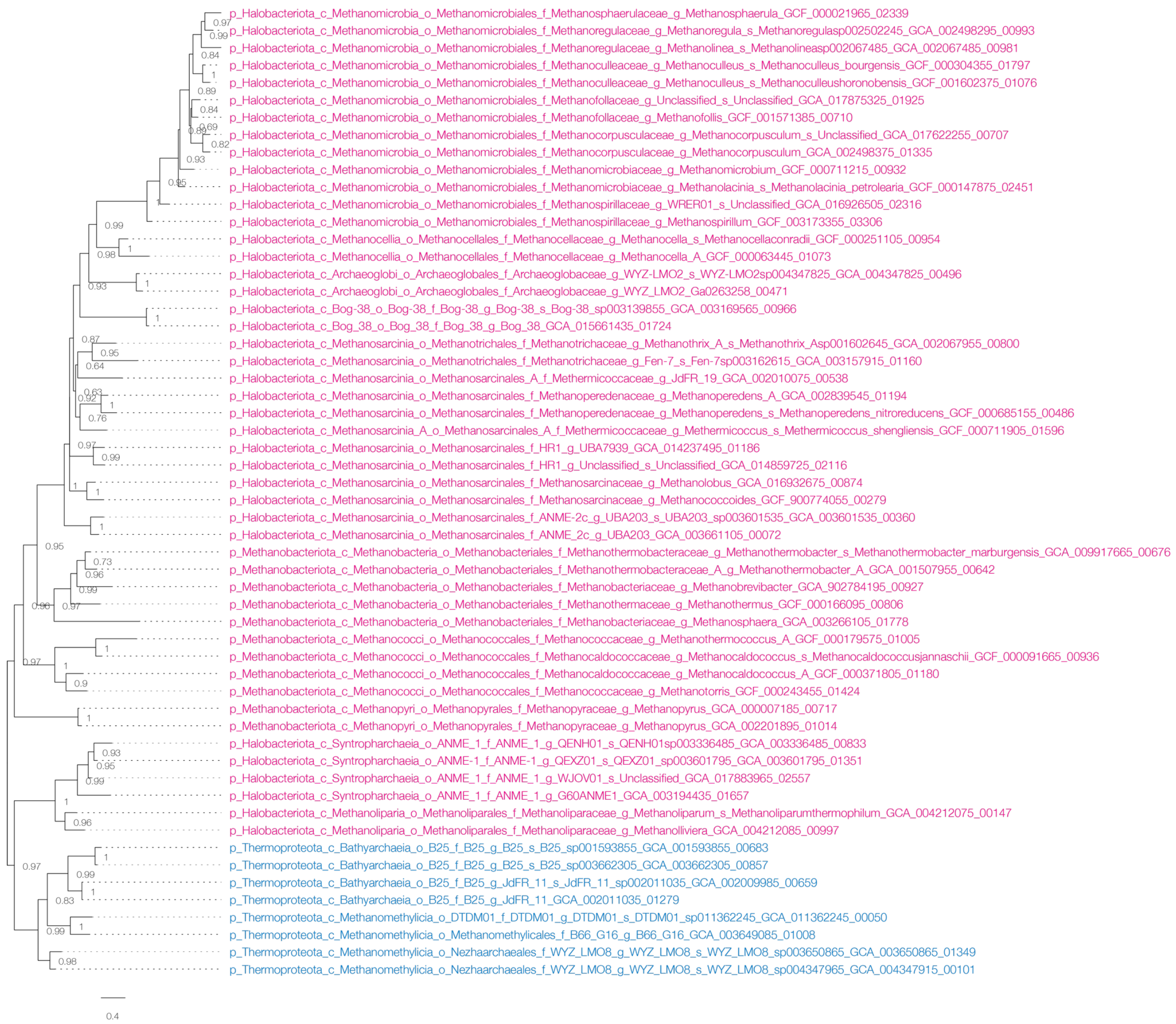

Figure S14. Phylogeny of MtrB for tetrahydromethanopterin S-methyltransferase subunit B. Maximum-likelihood tree is constructed using IQ-tree with LG+C10+G+F that is chosen as the best-fit model according to BIC. Values on nodes are tbe-transformed ultrafast bootstrap values. Taxa are colored according to Euryarchaeota (pink) and TACK (blue). TACK sequences (as well as some horizontally transferred Halobacteriota) are placed as basal divergence from most Euryarchaeota, supported by gene-species reconciliation. The datasets used to generate this tree (original and trimmed alignment) and the corresponding tree in Newick format are provided in Supplementary Datasets.

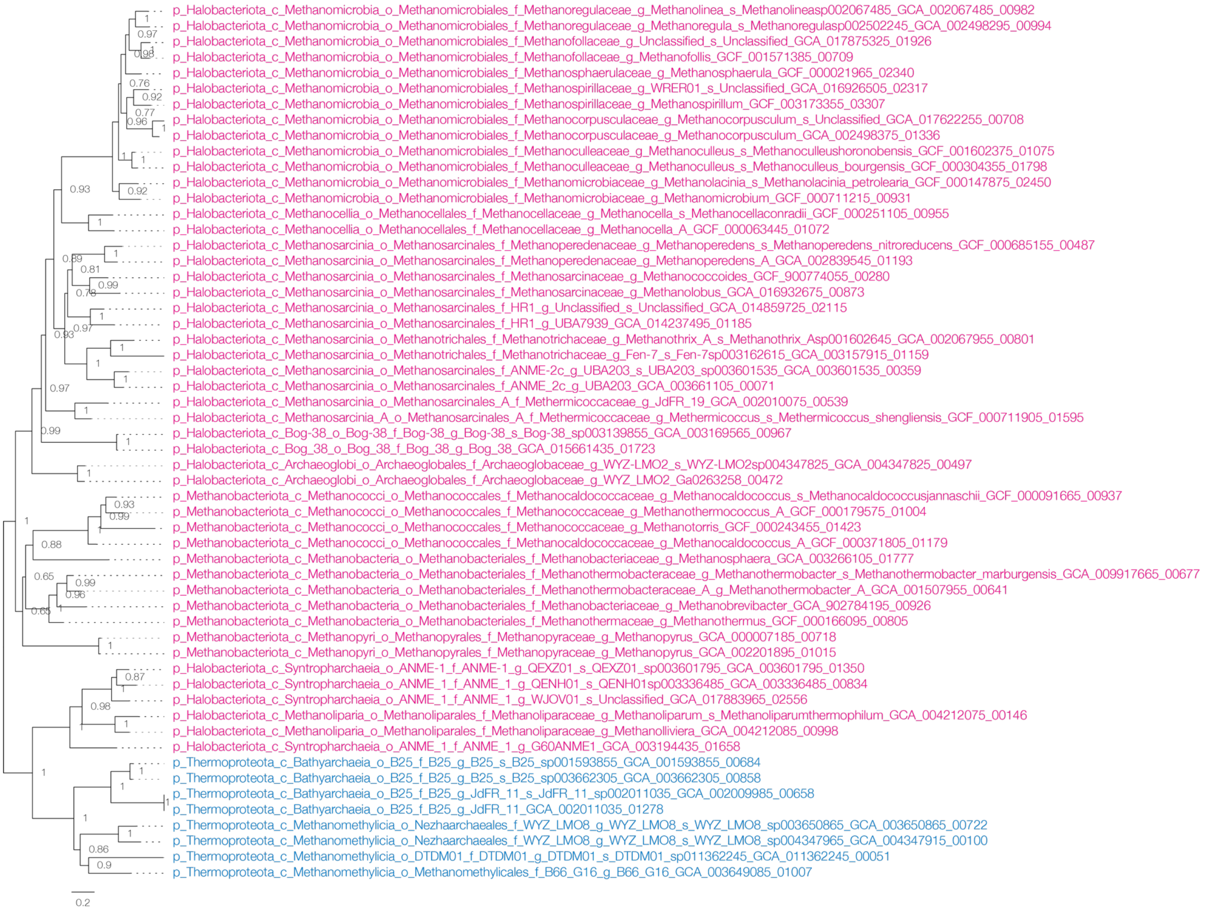

Figure S15. Phylogeny of MtrA for tetrahydromethanopterin S-methyltransferase subunit A. Maximum-likelihood tree is constructed using IQ-tree with LG+C30+G+F that is chosen as the best-fit model according to BIC. Values on nodes are tbe-transformed ultrafast bootstrap values. Taxa are colored according to Euryarchaeota (pink) and TACK (blue). TACK sequences (as well as some horizontally transferred Halobacteriota) are placed as basal divergence from most Euryarchaeota, supported by gene-species reconciliation. The datasets used to generate this tree (original and trimmed alignment) and the corresponding tree in Newick format are provided in Supplementary Datasets.

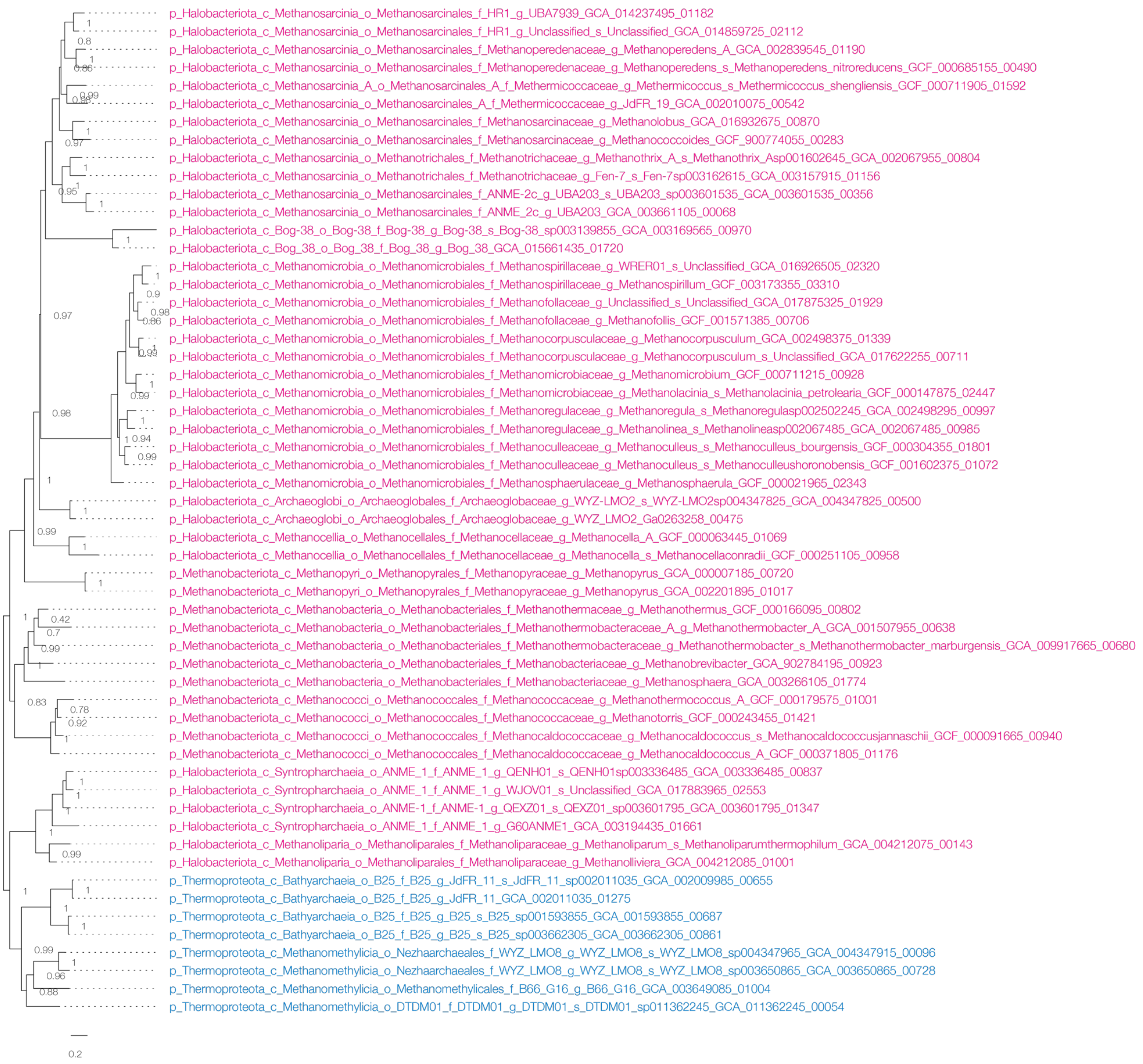

Figure S16. Phylogeny of MtrH for tetrahydromethanopterin S-methyltransferase subunit H. Maximum-likelihood tree is constructed using IQ-tree with LG+C30+G+F that is chosen as the best-fit model according to BIC. Values on nodes are tbe-transformed ultrafast bootstrap values. Taxa are colored according to Euryarchaeota (pink) and TACK (blue). TACK sequences (as well as some horizontally transferred Halobacteriota) are placed as basal divergence from most Euryarchaeota, supported by gene-species reconciliation. The datasets used to generate this tree (original and trimmed alignment) and the corresponding tree in Newick format are provided in Supplementary Datasets.

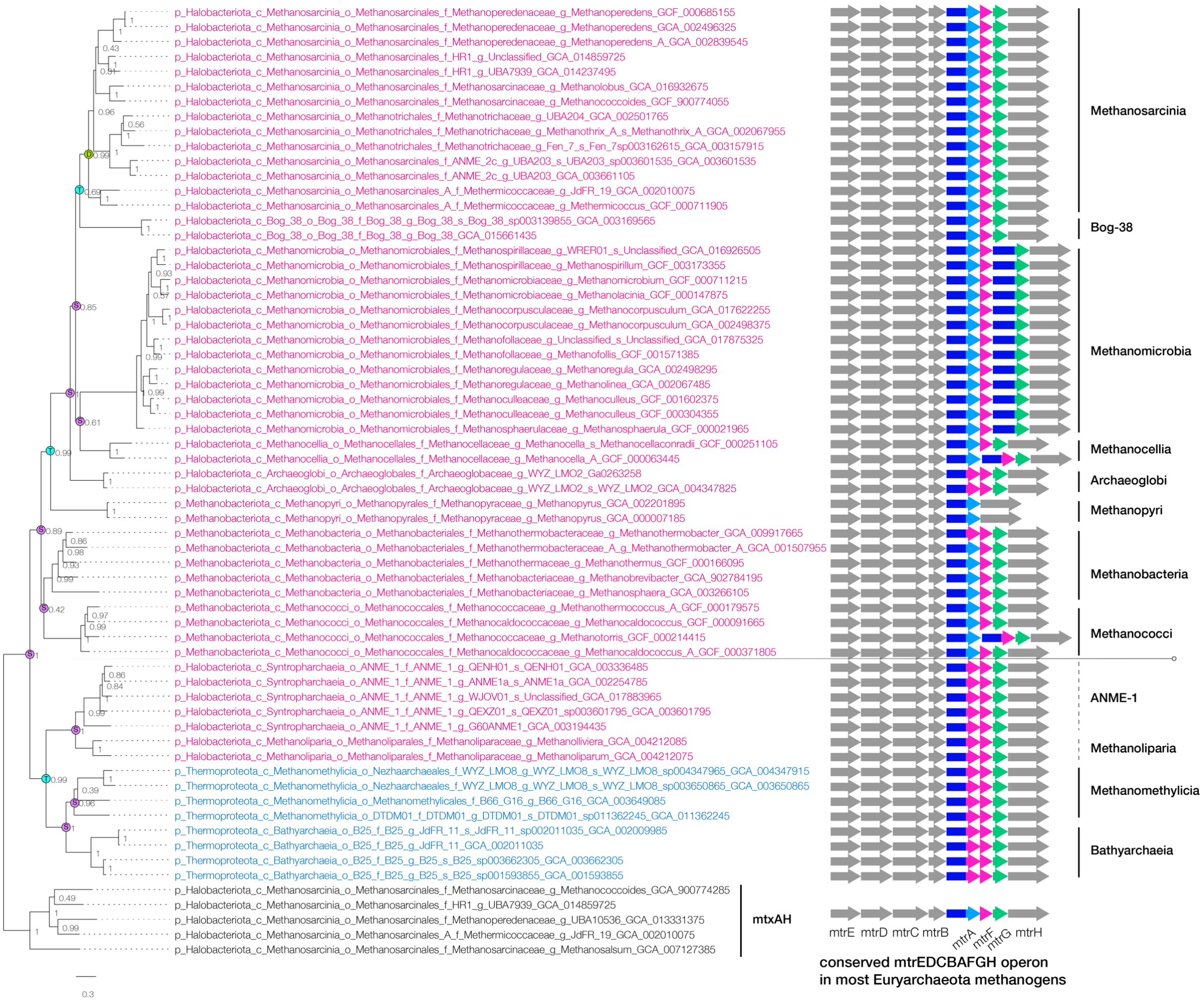

Figure S17. Phylogeny of MtrAH (uncollapsed version of Figure 1C). Bayesian inference was performed using PhyloBayes (-cat -gtr). The displayed tree is a consensus tree of two independent chains that converge after 5000 runs with a burn-in of 1000 (maxdiff<0.2). Values on nodes are posterior probabilities. Taxa are colored according to Euryarchaeota (pink) and TACK (blue). Sequences for MtxAH were used as the outgroup. With this outgroup, TACK sequences (as well as some horizontally transferred Halobacteriota) are placed as basal divergence from most Euryarchaeota. Schematic view of operon structure is displayed. Canonical mtrA is composed of a conserved N-terminal domain (colored in dark blue) and C-terminal transmembrane domain (colored in light blue). Some genomes have a fusion type containing a fused mtrA with its C-terminus replaced by a different transmembrane subunit of mtr operon (mtrF in pink or mtrG in green). Other subunits are colored in grey. Duplication (D), transfer (T), and speciation (S) events are denoted on deep nodes. The datasets used to generate this tree (original and trimmed alignment) and the corresponding tree in Newick format are provided in Supplementary Datasets.

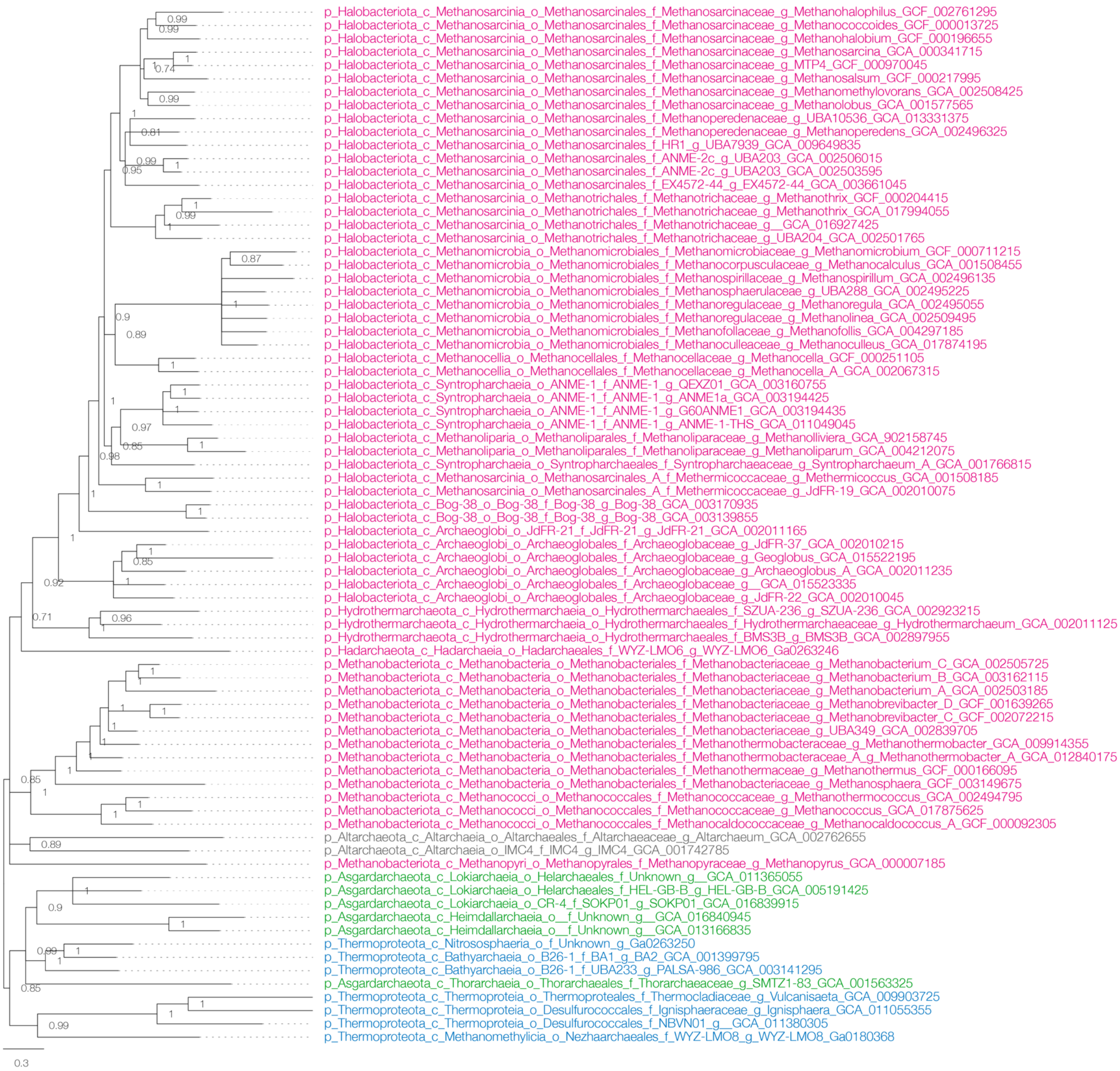

Figure S18. Phylogeny of MfnABDEF for methanofuran biosynthesis. Bayesian inference was performed using PhyloBayes (-cat -gtr). The displayed tree is a consensus tree of two independent chains that converge after 10000 runs with a burn-in of 1000 (maxdiff<0.2). Values at nodes are posterior probabilities. Taxa are colored according to Euryarchaeota (pink), TACK (blue), DPANN (purple), and Asgardarchaeota (green). TACK and Asgardarchaeota, despite polytomy and horizontal transfers, collectively form a sistering cluster with Euryarchaeota. The datasets used to generate this tree (original and trimmed alignment) and the corresponding tree in Newick format are provided in Supplementary Datasets.

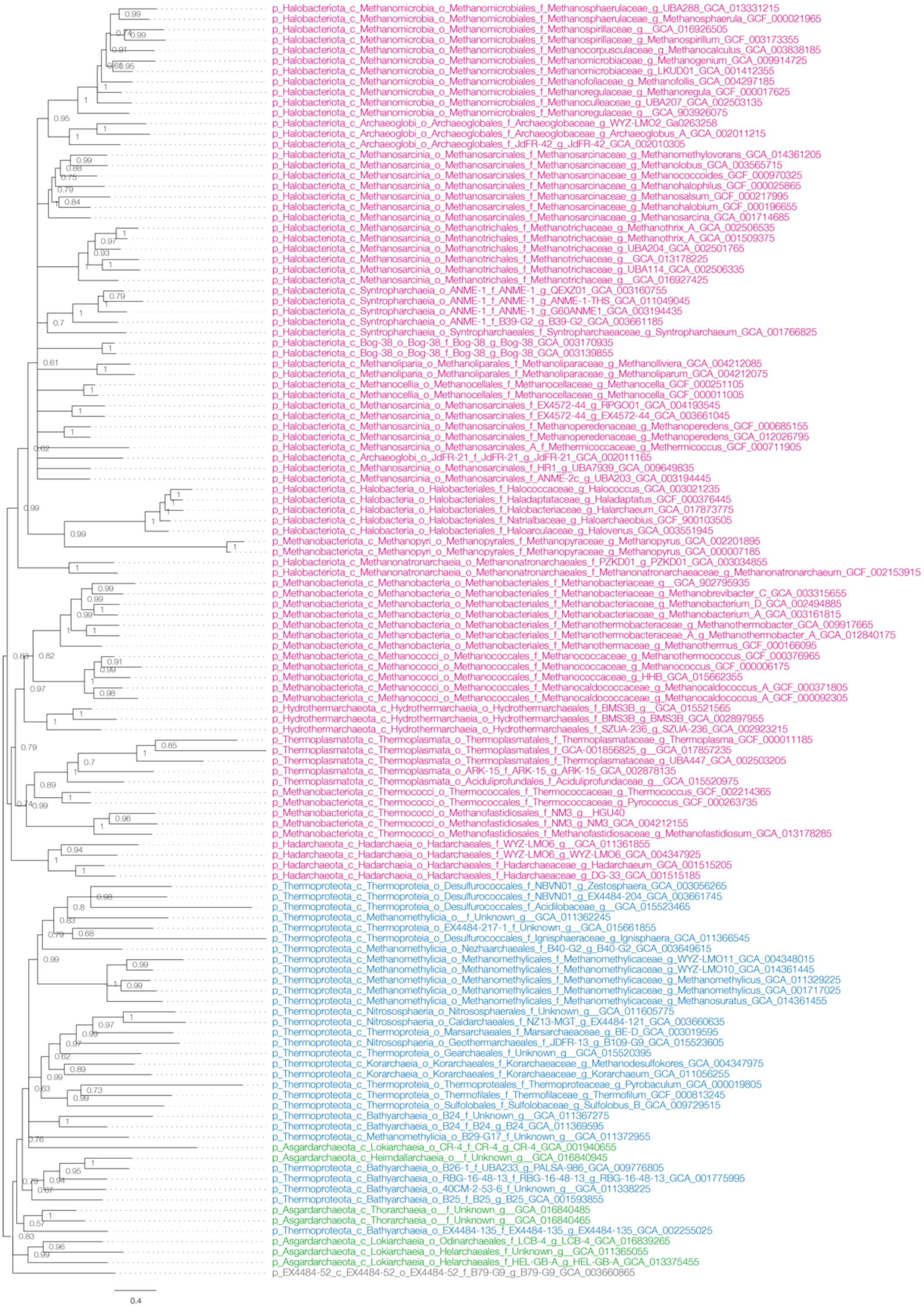

Figure S19. Phylogeny of MoaABC for molybdopterin biosynthesis. Bayesian inference was performed using PhyloBayes (-cat -gtr). The displayed tree is a consensus tree of two independent chains that converge after 10000 runs with a burn-in of 1000 (maxdiff<0.2). Values at nodes are posterior probabilities. Taxa are colored according to Euryarchaeota (pink), TACK (blue), and Asgardarchaeota (green). TACK and Asgardarchaeota, despite polytomy and horizontal transfers, collectively form a sistering cluster with Euryarchaeota. The datasets used to generate this tree (original and trimmed alignment) and the corresponding tree in Newick format are provided in Supplementary Datasets.

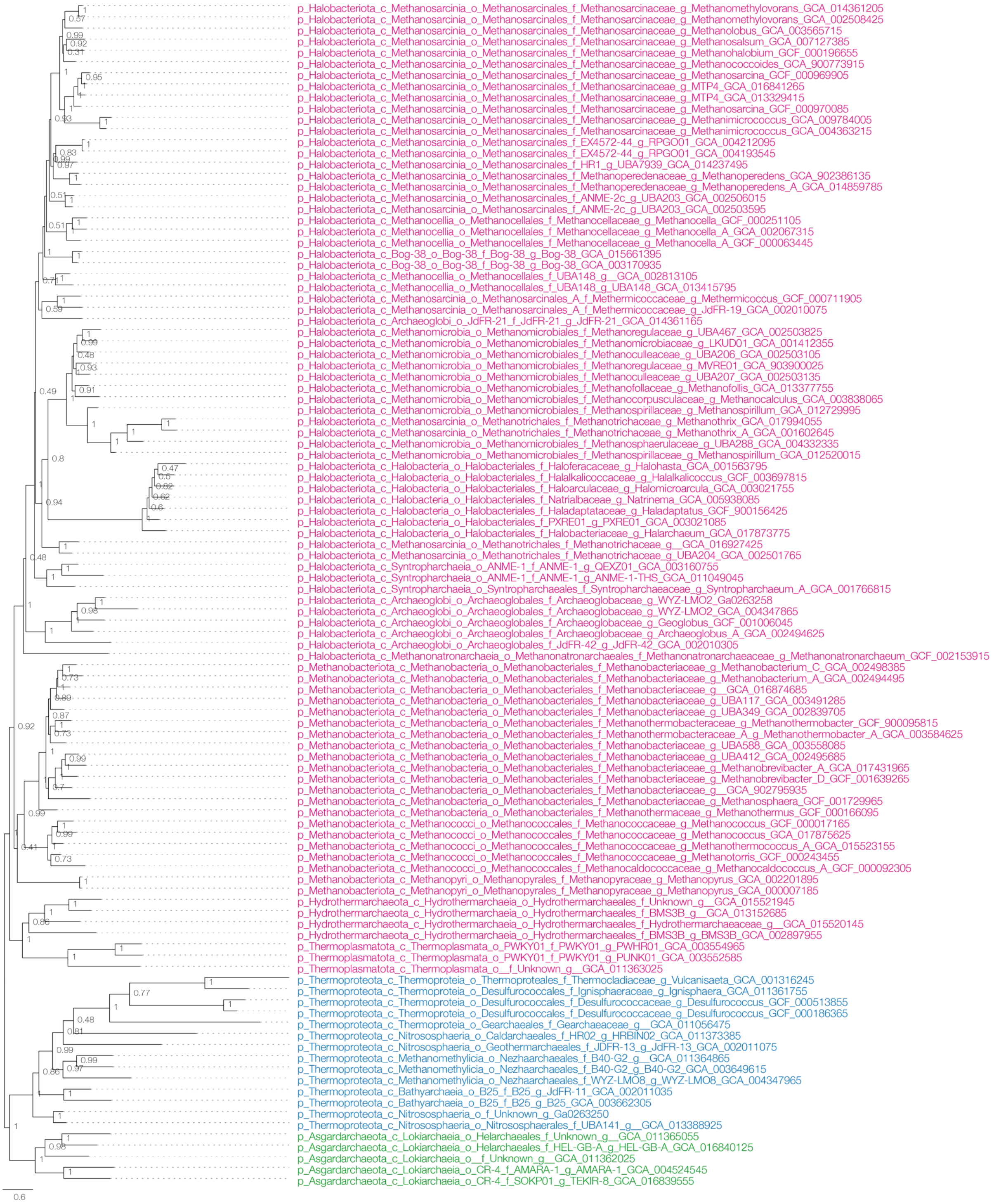

Figure S20. Phylogeny of MptADEG for methanopterin biosynthesis. Bayesian inference was performed using PhyloBayes (-cat -gtr). The displayed tree is a consensus tree of two independent chains that converge after 10000 runs with a burn-in of 1000 (maxdiff<0.2). Values at nodes are posterior probabilities. Taxa are colored according to Euryarchaeota (pink), TACK (blue), and Asgardarchaeota (green). TACK and Asgardarchaeota collectively form a sistering cluster with Euryarchaeota. The datasets used to generate this tree (original and trimmed alignment) and the corresponding tree in Newick format are provided in Supplementary Datasets.

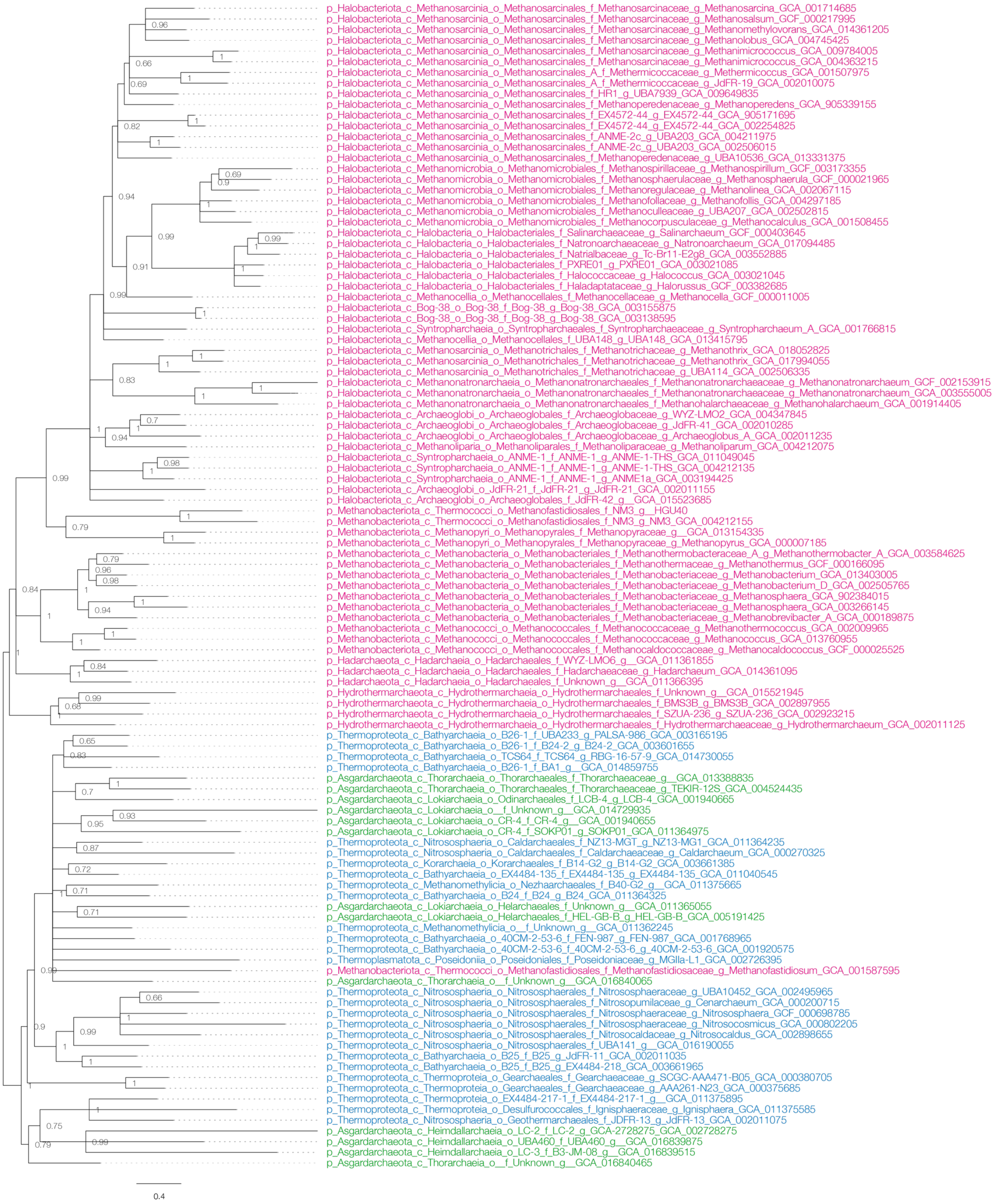

Figure S21. Phylogeny of CofCD for cofactor F420 biosynthesis. Bayesian inference was performed using PhyloBayes (-cat -gtr). The displayed tree is a consensus tree of two independent chains that converge after 10000 runs with a burn-in of 1000 (maxdiff<0.2). Values at nodes are posterior probabilities. Taxa are colored according to Euryarchaeota (pink), TACK (blue), and Asgardarchaeota (green). TACK and Asgard, despite polytomy and frequent horizontal transfers, collectively forms a sistering cluster with Euryarchaeota. The datasets used to generate this tree (original and trimmed alignment) and the corresponding tree in Newick format are provided in Supplementary Datasets.

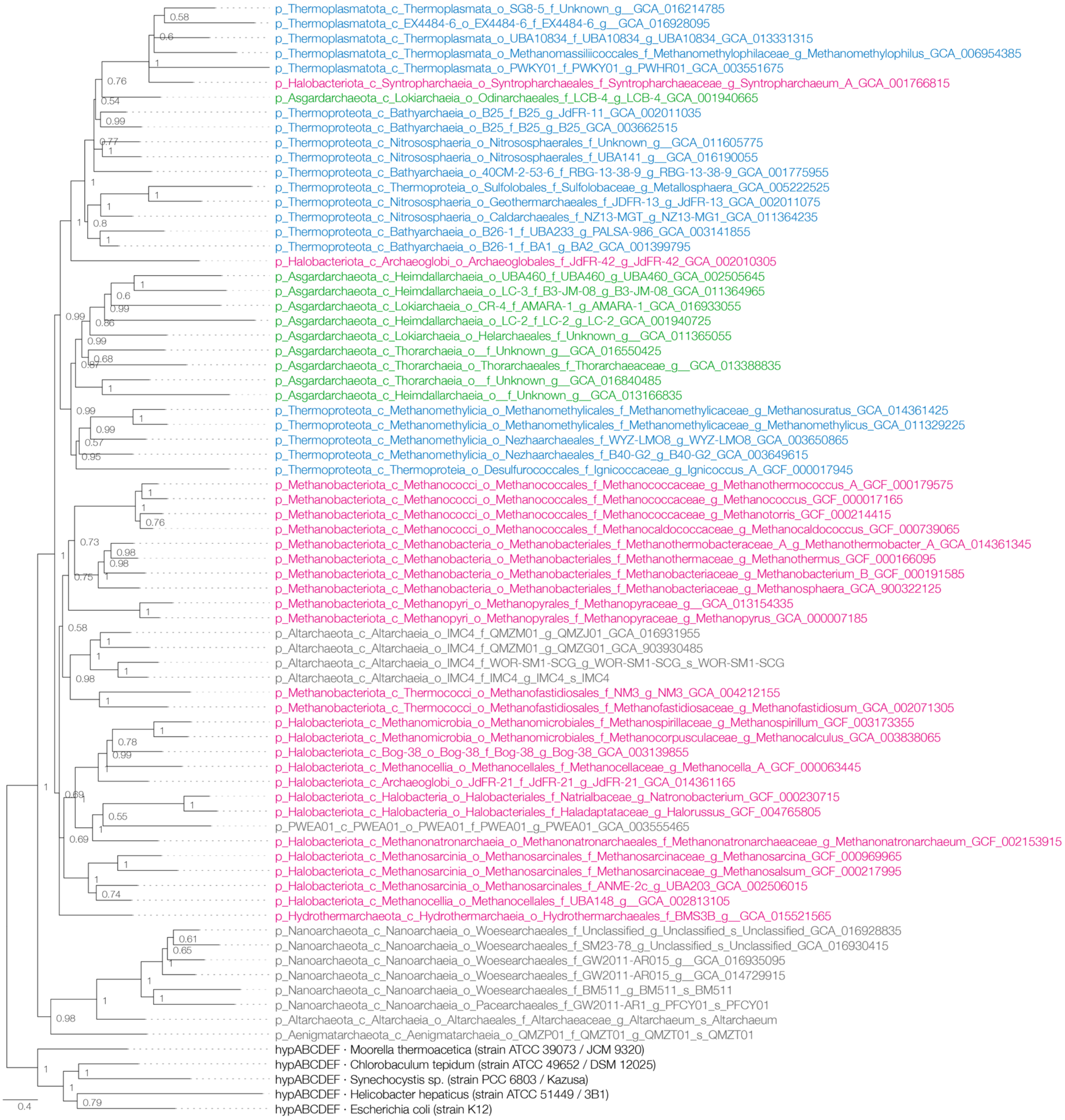

Figure S22. Phylogeny of HypABCDEF for [NiFe] hydrogenase maturation. Bayesian inference was performed using PhyloBayes (-cat -gtr). The displayed tree is a consensus tree of two independent chains that converge after 10000 runs with a burn-in of 1000 (maxdiff<0.2). Values at nodes are posterior probabilities. Taxa are colored according to Euryarchaeota (pink), TACK (blue), Asgardarchaeota (green), and DPANN (purple). The datasets used to generate this tree (original and trimmed alignment) and the corresponding tree in Newick format are provided in Supplementary Datasets.

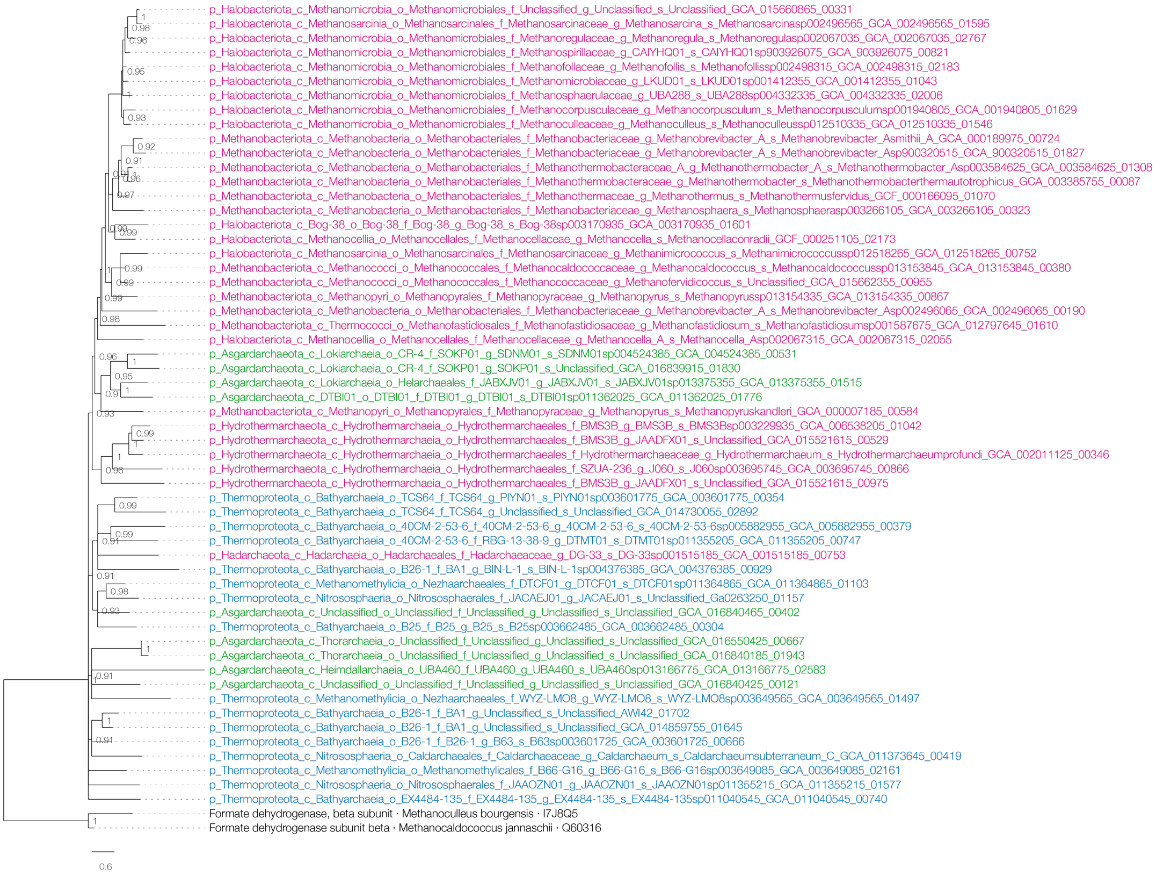

Figure S23. Phylogeny of FrhB for coenzyme F_{420} hydrogenase subunit beta. Maximum-likelihood tree is constructed using IQ-tree with LG+C40+G+F that is chosen as the best-fit model according to BIC. Values on nodes are tbe-transformed ultrafast bootstrap values. Nodes with <90% support are collapsed. Taxa are colored according to Euryarchaeota (pink), TACK (blue), and Asgardarchaeota (green). Sequences for formate dehydrogenase subunit B are used as the outgroup. Despite polytomy, TACK and Asgardarchaeota sequences are separated from Euryarchaeota. The datasets used to generate this tree (original and trimmed alignment) and the corresponding tree in Newick format are provided in Supplementary Datasets.

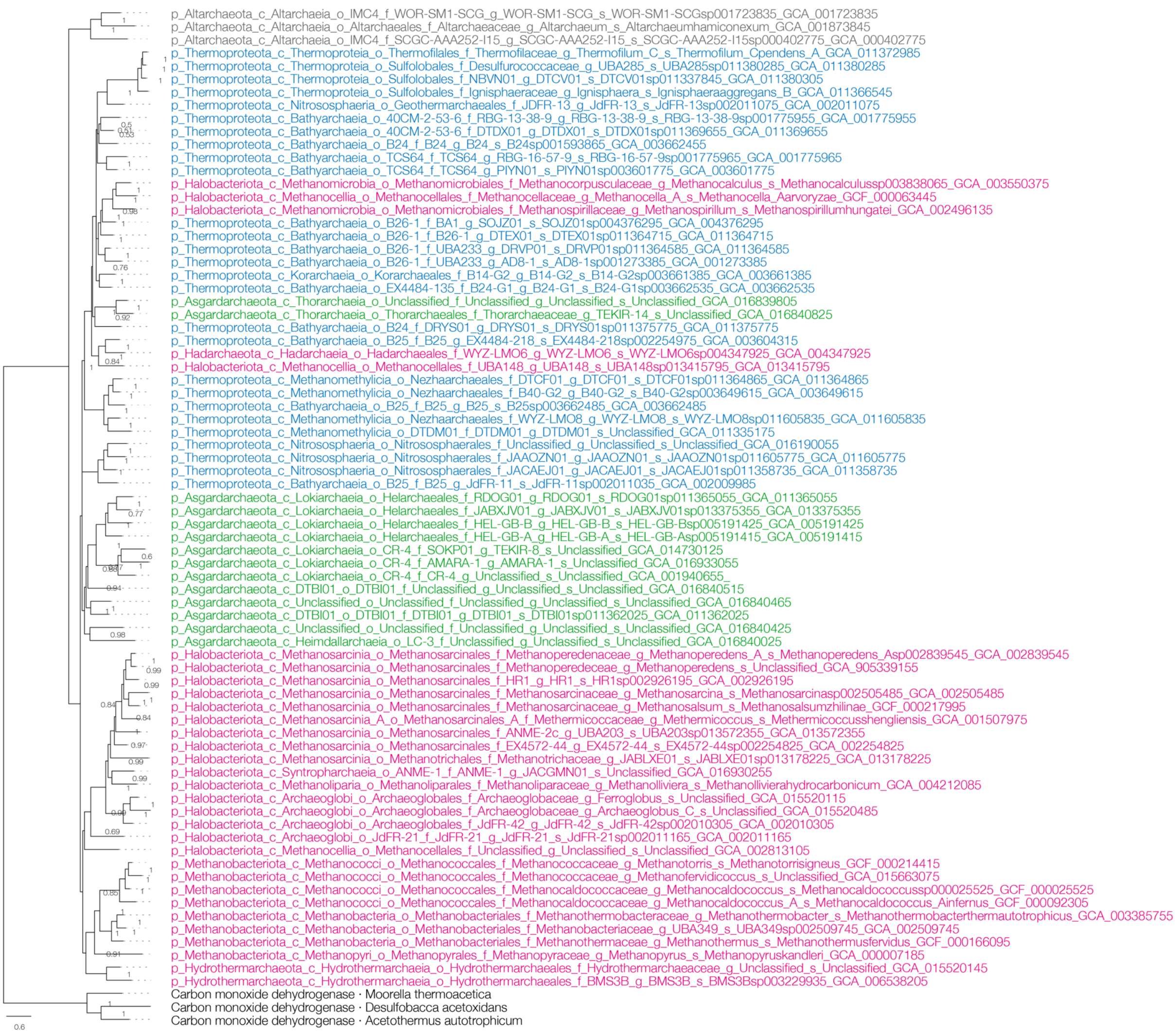

Figure S24. Phylogeny of Acs/CodhACDE for acetyl-CoA synthase/carbon monoxide dehydrogenase. Bayesian inference was performed using PhyloBayes (-cat -gtr). The displayed tree is a consensus tree of two independent chains that converge after 10000 runs with a burn-in of 1000 (maxdiff<0.2). Values at nodes are posterior probabilities. Taxa are colored according to Euryarchaeota (pink), TACK (blue), Asgardarchaeota (green), and DPANN (purple). Bacterial homologs are used as outgroup. The datasets used to generate this tree (original and trimmed alignment) and the corresponding tree in Newick format are provided in Supplementary Datasets.

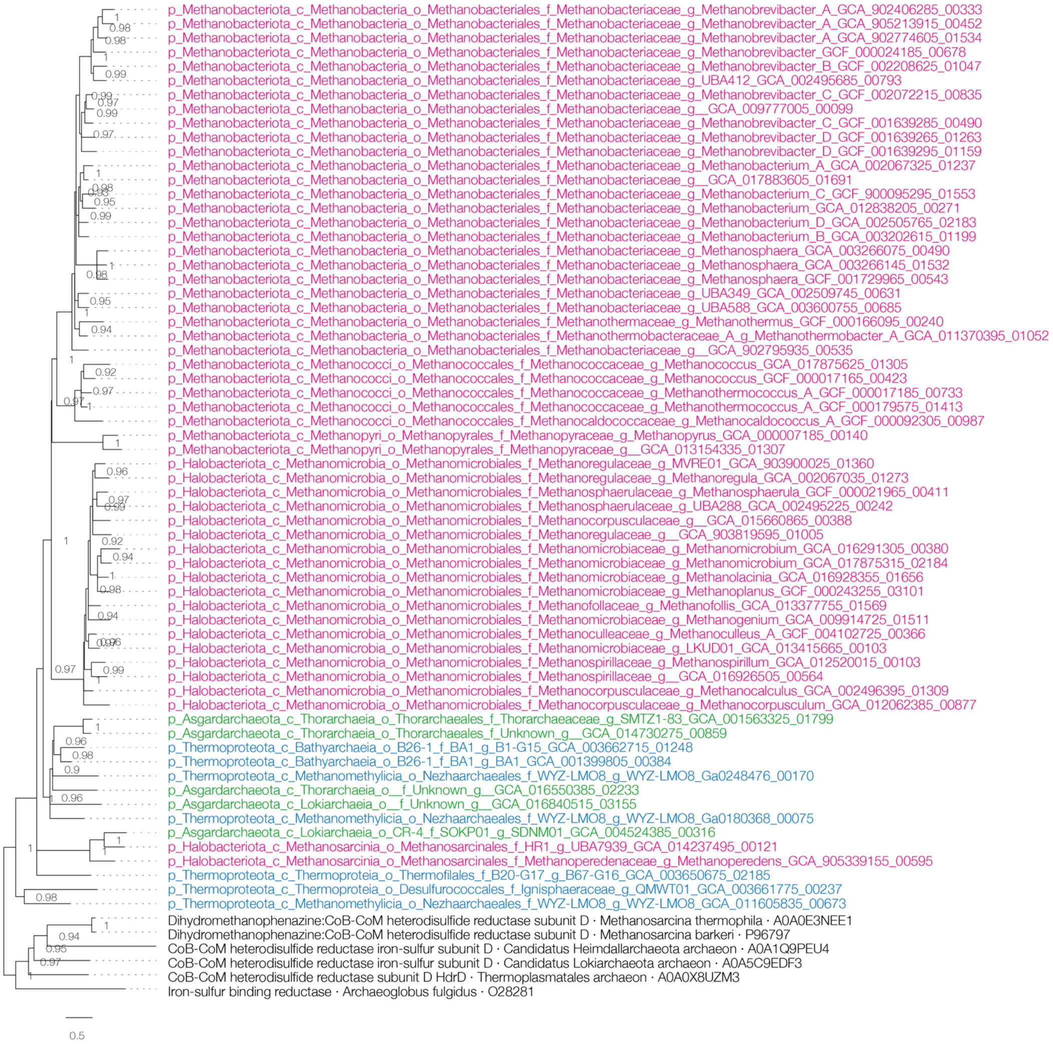

Figure S25. Phylogeny of TfrB for thiol:fumarate reductase. Maximum-likelihood tree is constructed using IQ-tree with LG+C40+G+F that is chosen as the best-fit model according to BIC. Values on nodes are tbe-transformed ultrafast bootstrap values. Nodes with <90% support are collapsed. Taxa are colored according to Euryarchaeota (pink), TACK (blue), and Asgardarchaeota (green). Sequences for HdrD are used as the outgroup. The datasets used to generate this tree (original and trimmed alignment) and the corresponding tree in Newick format are provided in Supplementary Datasets.

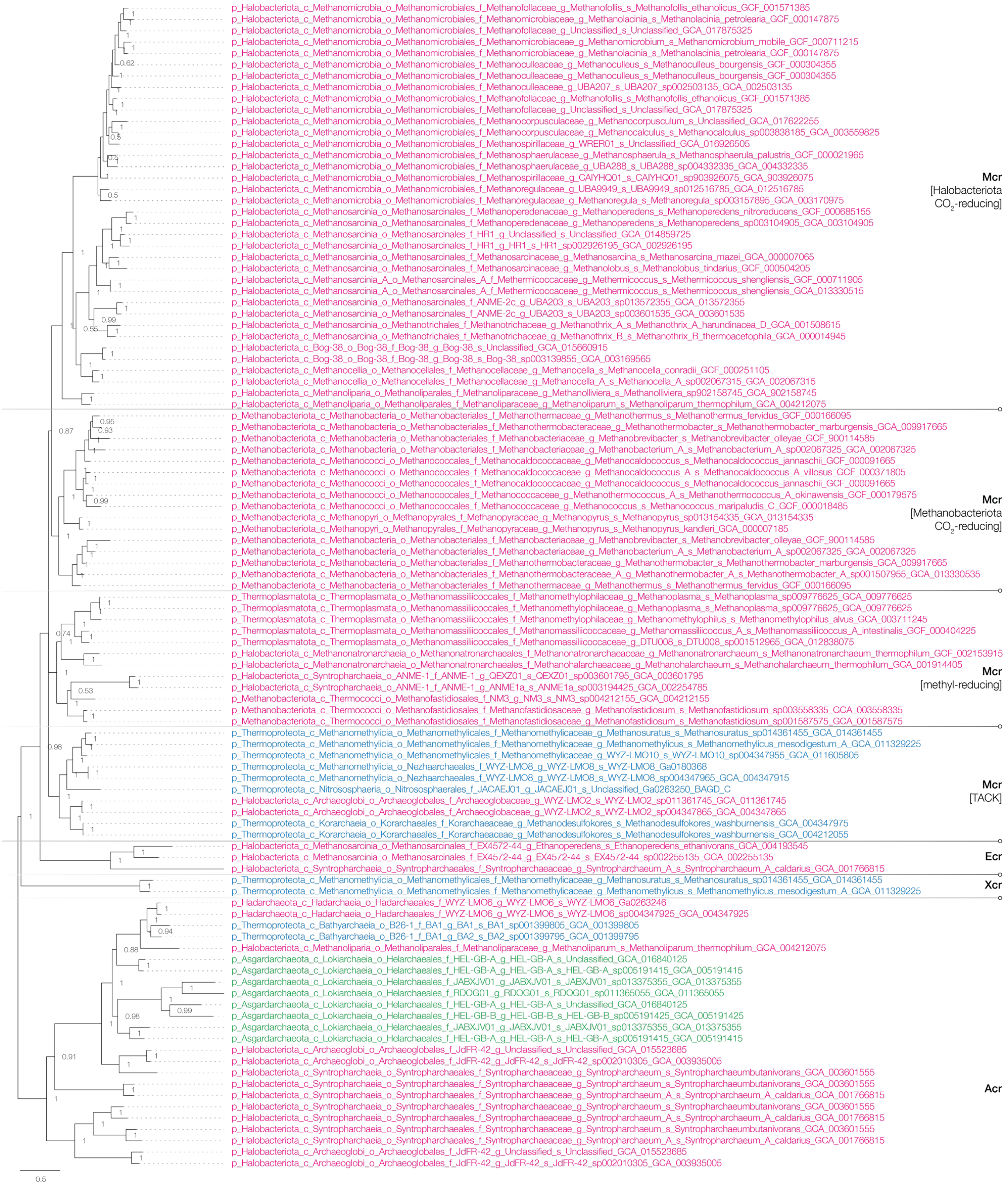

Figure S26. Phylogeny of McrBGA and homologs. Bayesian inference was performed using PhyloBayes (-cat -gtr). The displayed tree is a consensus tree of two independent chains that converge after 5000 runs with a burn-in of 1000 (maxdiff<0.2). Values at nodes are posterior probabilities. Taxa are colored according to Euryarchaeota (pink), TACK (blue), and Asgardarchaeota (green). The datasets used to generate this tree (original and trimmed alignment) and the corresponding tree in Newick format are provided in Supplementary Datasets.

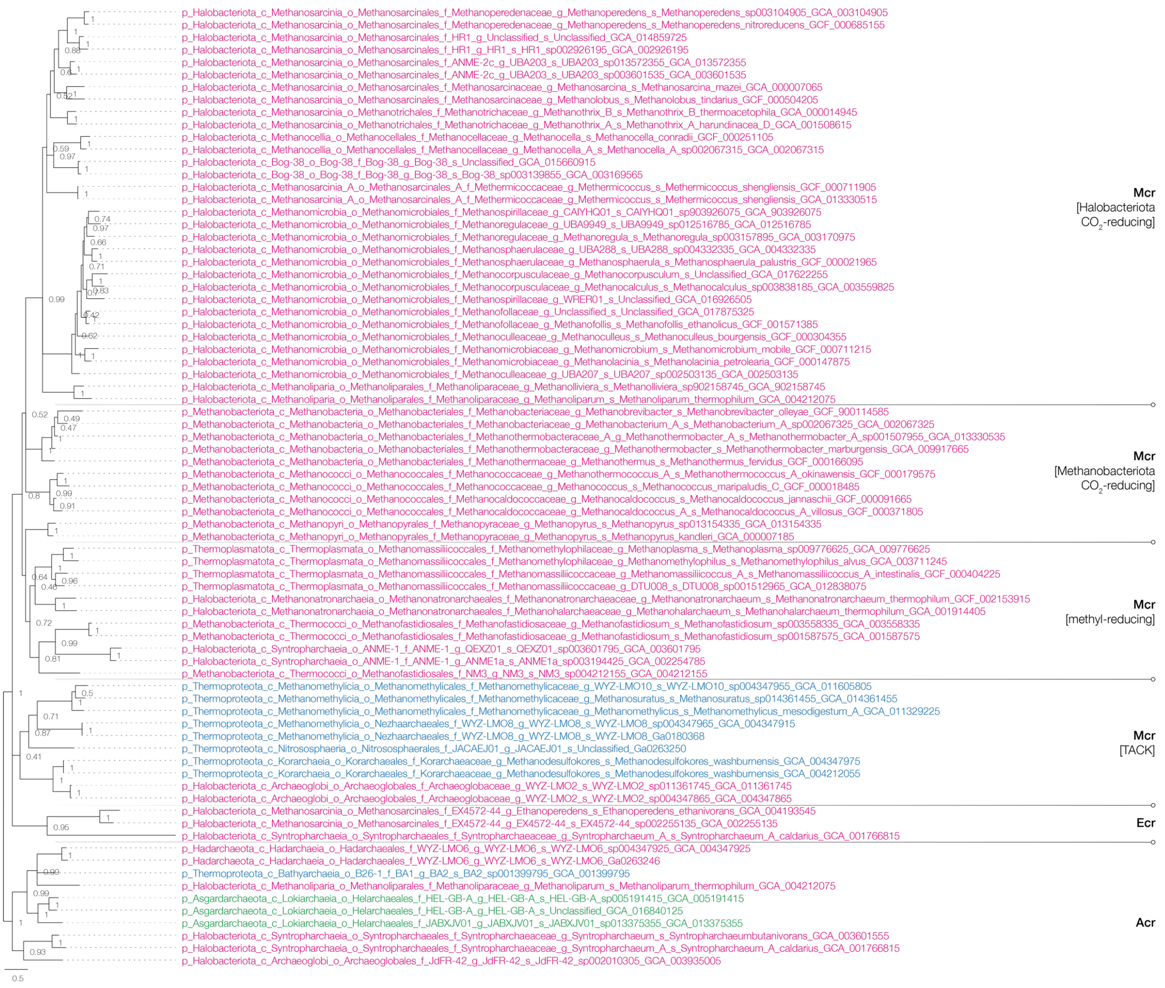

Figure S27. Phylogeny of McrCD and homologs. Bayesian inference was performed using PhyloBayes (-cat -gtr). The displayed tree is a consensus tree of two independent chains that converge after 5000 runs with a burn-in of 1000 (maxdiff<0.2). Values at nodes are posterior probabilities. Taxa are colored according to Euryarchaeota (pink), TACK (blue), and Asgardarchaeota (green). We designate association with a specific alkyl-S-CoM reductase (*e.g.*, Mcr, Ecr, and Acr) according to proteins C/D that have unambiguous associated BGA (*i.e.*, in the same operon with BGA or only one set of BGA and CD are present in the genome). The datasets used to generate this tree (original and trimmed alignment) and the corresponding tree in Newick format are provided in Supplementary Datasets.

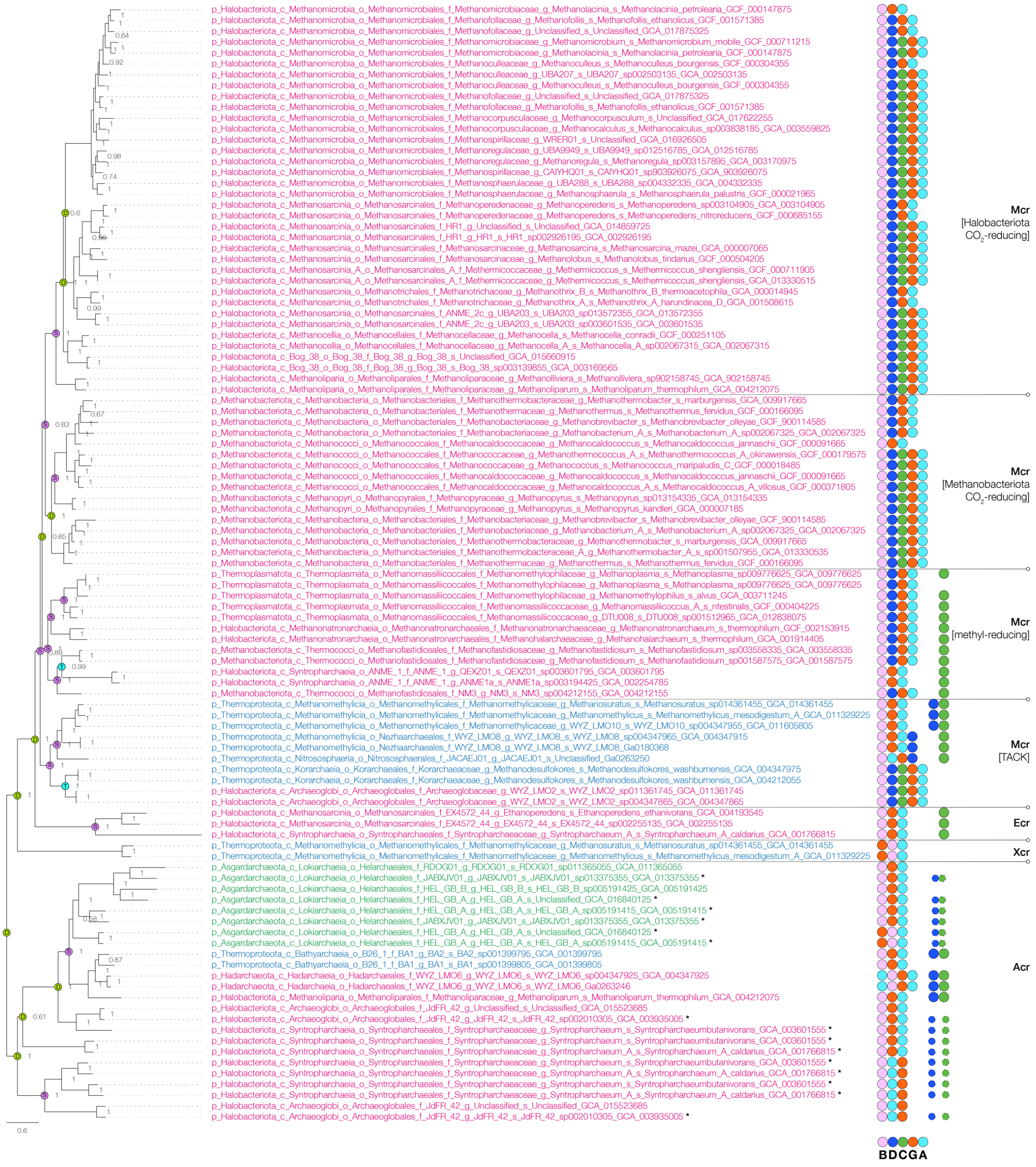

Figure S28. Phylogeny of McrBDCGA and homologs (uncollapsed version of Figure 1D). The concatenation of BGA and CD sequences is unequivocal (solid-line circles on the right-hand side of the figure) if (i) they are on the same operon, (ii) BGA and C/D are disconnected but there is only one copy of BGA and C/D each in the genome, and (iii) proteins C/D are clearly associated one of the BGA sets (*e.g.*, if McrBGA and AcrBGA are present and C/D classified as AcrCD if Fig. S27, then concatenate AcrBGACD). The association between proteins C/D and BGA is ambiguous only for a few Helarchaeales, Syntrophoarchaeum, and Archaeoblobales (indicated with *) that have multiple AcrBGA but one AcrCD. In these cases, proteins C/D are not concatenated with any BGA and left as gaps in the alignment. In the figure, these proteins C/D are shown as small dotted-line circles along with all AcrBGA present in that genome. Overall, the Mcr cluster has a unique BDCGA operon, which is also ancestral because it is highly conserved and is found in basal lineages such as Methanodesulfokores. Noteworthy is the entire methyl-

reducing Mcr subcluster has a BDGA operon with a discrete gene C sometimes in the vicinity of the main operon. Such structure is also sporadically observed within Methanobacteriota, Halobacteriota, and TACK, suggesting that it is likely an independently derived variance from the BDCGA operon. Clusters other than Mcr do not have the BDCGA or BDGA operon. While it is difficult to infer the exact operon structure of the organism that possessed the ancestral methyl/alkyl-CoM reductase, this organism should have all the five genes because genomes that miss C and/or D (*e.g.*, ANME-1, EX4572-44, and BA1) are sporadic and not basal in the tree. Bayesian inference was performed using PhyloBayes (-cat -gtr). The displayed tree is a consensus tree of two independent chains that converge after 5000 runs with a burn-in of 1000 (maxdiff<0.2). Values at nodes are posterior probabilities. Taxa are colored according to Euryarchaeota (pink), TACK (blue), and Asgardarchaeota (green). Duplication (D), transfer (T), and speciation (S) events are denoted on deep nodes. The datasets used to generate this tree (original and trimmed alignment) and the corresponding tree in Newick format are provided in Supplementary Datasets.

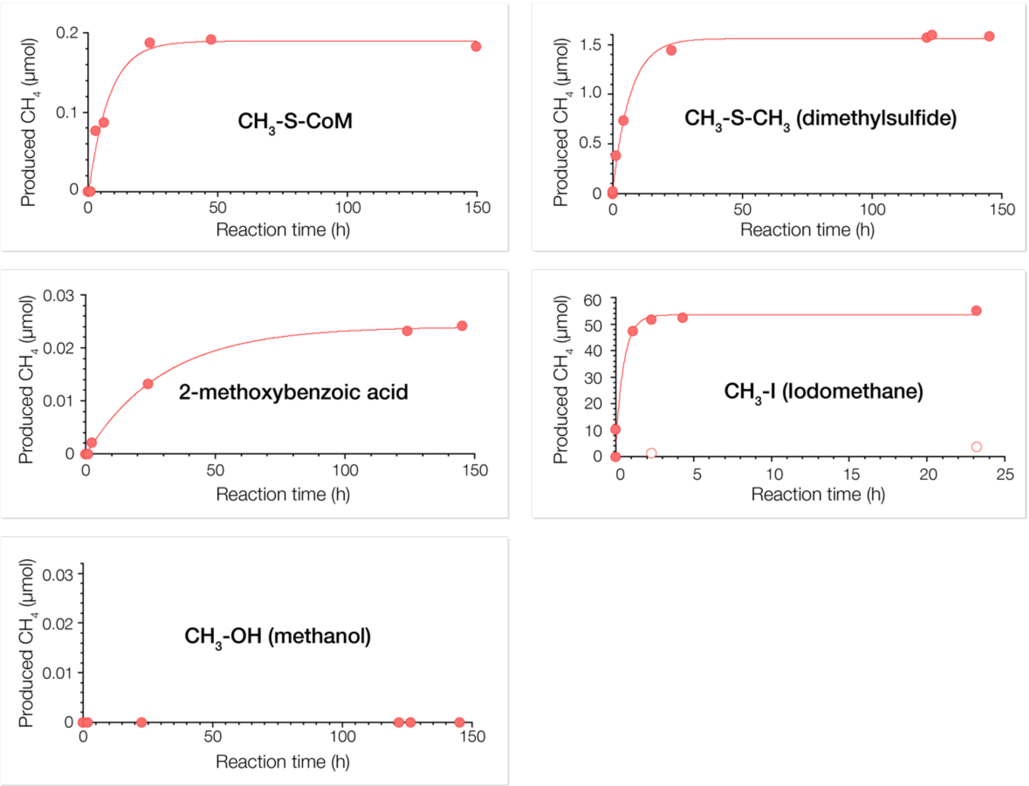

Figure S29. Autocatalysis of F_{430} on methylated compounds. Incubation of F_{430} with methylated compounds lead to methane production except for methanol. For control incubations without F_{430}, methane was only detected in treatments containing iodomethane (open circles).

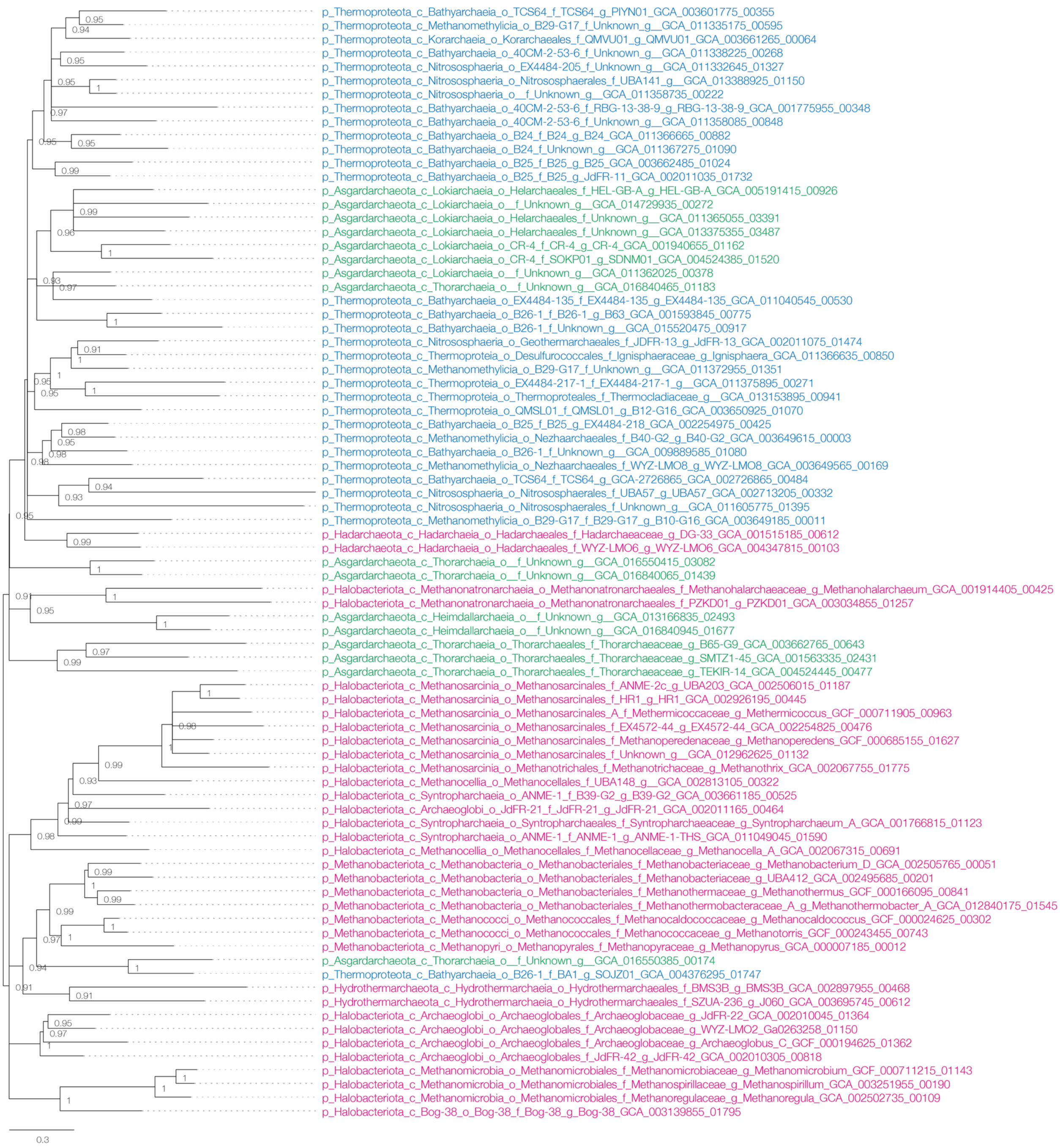

Figure S30. Phylogeny of Mtd for F_{420}-dependent methylene-H_4MPT dehydrogenase. Maximum-likelihood tree is constructed using IQ-tree with LG+C40+G+F that is chosen as the best-fit model according to BIC. Values on nodes are tbe-transformed ultrafast bootstrap values. Nodes with <90% support are collapsed. Taxa are colored according to Euryarchaeota (pink), TACK (blue), and Asgardarchaeota (green). Despite polytomy and horizontal transfers, TACK and Asgardarchaeota sequences are separated from the majority of Euryarchaeota. The datasets used to generate this tree (original and trimmed alignment) and the corresponding tree in Newick format are provided in Supplementary Datasets.

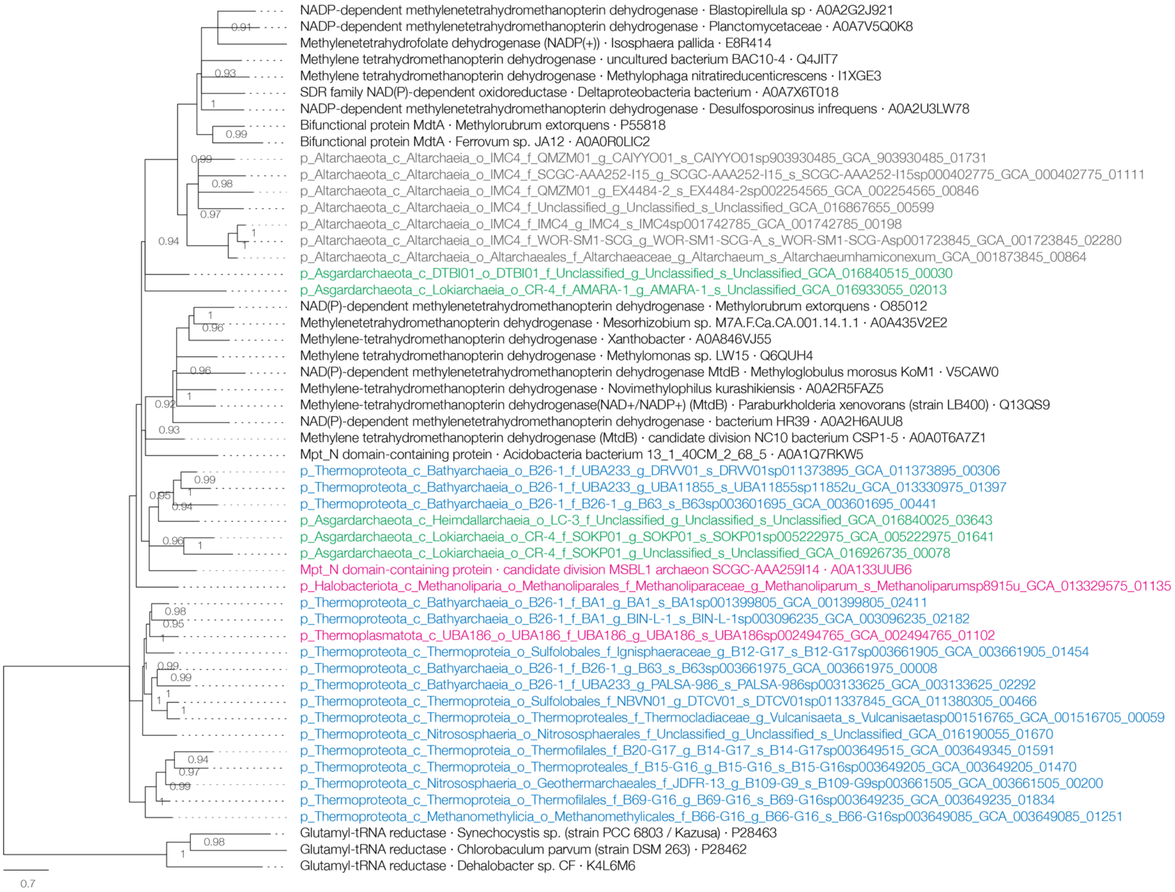

Figure S31. Phylogeny of N-Mtd for NAD(P)H-dependent methylene-H_4MPT dehydrogenase. Maximum-likelihood tree is constructed using IQ-tree with LG+C40+G+F that is chosen as the best-fit model according to BIC. Values on nodes are tbe-transformed ultrafast bootstrap values. Nodes with <90% support are collapsed. Taxa are colored according to Euryarchaeota (pink), TACK (blue), Asgardarchaeota (green), DPANN (purple), and Bacteria (black). Bacterial glutamyl-tRNA reductase is used as outgroup. The datasets used to generate this tree (original and trimmed alignment) and the corresponding tree in Newick format are provided in Supplementary Datasets.

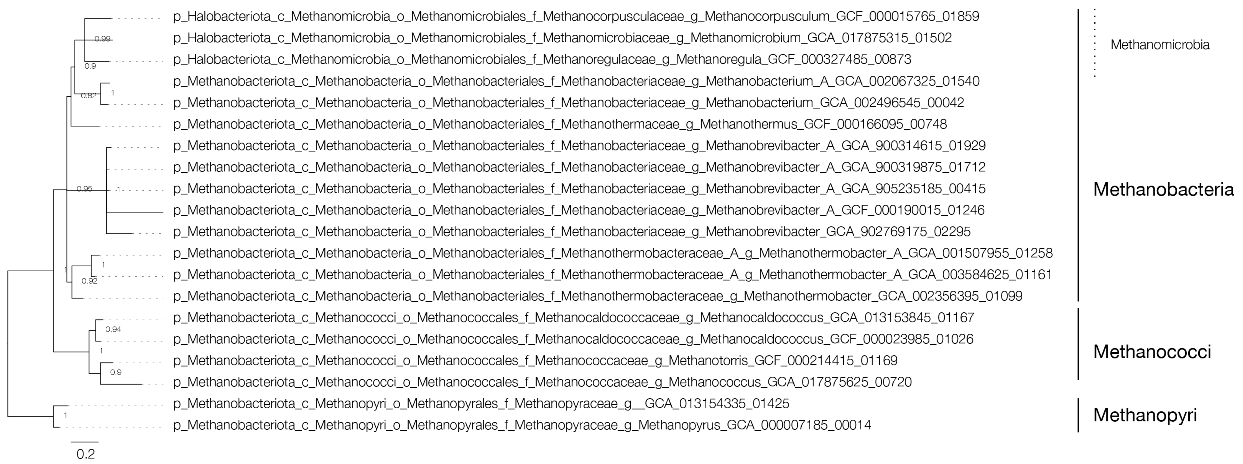

Figure S32. Phylogeny of Hmd for H_2-dependent methylene-H_4MPT dehydrogenase. Maximum-likelihood tree is constructed using IQ-tree with LG+C40+G+F that is chosen as the best-fit model according to BIC. Values on nodes are tbe-transformed ultrafast bootstrap values. Nodes with <90% support are collapsed. The datasets used to generate this tree (original and trimmed alignment) and the corresponding tree in Newick format are provided in Supplementary Datasets.

Supplementary Tables

Table S1. Quasi-equilibrium calculation of CH3-S-CoM concentration.

Reaction and standard free energy change

	Reaction	dG0' (kJ/mol)	dGdis (kJ/mol)	dGinv (kJ/mol)	dG (kJ/mol)
		25°C, H2/CO2/CH4 10E5 Pa, pH 7.0, others 1 M	energy dissipation/harvest	energy investment	equilibrium
CO2 reduction	CO2+MFR+Fdred2-+2H+ = CHO-MFR+Fdox+H2O	0		30	30
	CHO-MFR+H4MPT = CHO-H4MPT+MFR	-5			0
	CHO-H4MPT+2H+ = CH~H4MPT++H2O	-5			0
	CH~H4MPT++F420H2 = CH2=H4MPT + F420 + H+	6			0
	CH2=H4MPT + F420H2 = CH3-H4MPT + F420	-6			0
	CH3-H4MPT + HS-CoM = CH3-S-CoM + H4MPT	-30	-40		-40
	CH3-S-CoM + HS-CoB = CH4 + CoM-S-S-CoB	-30	-5		-5
	2H2 + CoM-S-S-CoB + Fdox = HS-CoM + HS-CoB + Fdred2- + 2 H	-39	-5		-5
	H2 + F420 = F420H2	-11			0
	H2 + Fdox = Fdred2- + 2 H+	16		30	30
methanol reduction	CH3OH + HS-CoM = CH3-S-CoM + H2O	-27.5			0
	CH3-S-CoM + HS-CoB = CH4 + CoM-S-S-CoB	-30	-5		-5
	2H2 + CoM-S-S-CoB + Fdox = HS-CoM + HS-CoB + Fdred2- + 2 H	-39	-5		-5
	H2 + Fdox = Fdred2- + 2 H+	16		60	60

Fixed substrate concentration

Compound	Concentration	Unit
CO2	0.01	bar
H2	0.0001	bar
H+	0.0000001	M
methanol	0.01	M

e-carrier ratio

e-carriers	Ratio (considering theoretical eq. w H2)
Fdred/Fdox	2.84E-02
Fdred/Fdox	5.12E+03
F420H2/F420	8.46E-03

Constant

F	96.485	kC/mole
R	0.008314	kJ/mol K
T	298.15	K
RT	2.4788191	kJ/mol

Cofactor total concentration

Cofactor	Value	Unit	Ref	after conversion (M)
MFR	1	nmol/mg protein	Daniels, 1993	2.40E-04
	6	nmol/mg protein	Daniels, 1993	1.44E-03
	0.7	nmol/mg cell DW	Jones et al., 1985	5.11E-04
	3.1	nmol/mg cell DW	Jones et al., 1985	2.26E-03
	2	nmol/mg cell DW	Poorter et al., 2003	1.46E-03
H4MPT	1.3	nmol/mg protein	Jones et al., 1985	3.12E-04
	3	nmol/mg protein	Jones et al., 1985	7.20E-04
	9.6	nmol/mg protein	Jones et al., 1985	2.30E-03
CoM	3	nmol/mg protein	Daniels, 1993	7.20E-04
	12	nmol/mg protein	Daniels, 1993	2.88E-03
	14.8	mM	Dybas et al., 1989	1.48E-02
CoB	1.42	nmol/mg cell DW	Poorter et al., 2003	1.04E-03
	1.61	nmol/mg cell DW	Poorter et al., 2003	1.18E-03
	2.01	nmol/mg cell DW	Poorter et al., 2003	1.47E-03
	1	nmol/mg protein	Jones et al., 1985	2.40E-04
				values in red are used

Conversion

	Value	Unit	Ref
protein vs. cell volume	0.24	g/mL	Milo, 2013
cell volume vs. dry weight	1.37	ul/mg	Dybas et al., 1989

Table S2. Biosynthetic capacity and growth rate predicted from genome

	Ave. number of amino acids and cofactors that can be synthesized	Ave. doubling time (h)
DPANN	3.17 ± 3.55	9.10 ± 10.31
Asgardarchaeota	9.87 ± 4.51	11.81 ± 5.12
TACK	20.43 ± 6.96	6.83 ± 6.80
Euryarchaeota	26.68 ± 6.19	6.07 ± 4.20

Dataset S1 (separate file). A summary table containing the genome accession and counts of identified genes is provided as supplementary data.

For or all phylogenetic trees, the original and trimmed alignments and the corresponding trees in Newick format are available at https://github.com/meiranmeiran/archaeaDataset.

Supplementary References

1. K. Decker, K. Jungermann, R. Thauer, Energy production in anaerobic organisms. *Angewandte Chemie International Edition in English* **9**, 138-158 (1970).
2. W. E. Balch, L. J. Magrum, G. E. Fox, R. S. Wolfe, C. R. Woese, An ancient divergence among the bacteria. *Journal of Molecular Evolution* **9**, 305-311 (1977).
3. G. E. Fox, L. J. Magrum, W. E. Balch, R. S. Wolfe, C. R. Woese, Classification of methanogenic bacteria by 16S ribosomal RNA characterization. *Proceedings of the National Academy of Sciences* **74**, 4537-4541 (1977).
4. C. R. Woese, L. J. Magrum, G. E. Fox, Archaebacteria. *Journal of molecular evolution* **11**, 245-252 (1978).
5. P. N. Evans *et al.*, An evolving view of methane metabolism in the Archaea. *Nature Reviews Microbiology* **17**, 219-232 (2019).
6. É. Bapteste, C. Brochier, Y. Boucher, Higher-level classification of the Archaea: evolution of methanogenesis and methanogens. *Archaea* **1**, 353-363 (2005).
7. S. Gribaldo, C. Brochier-Armanet, The origin and evolution of Archaea: a state of the art. *Philosophical Transactions of the Royal Society B: Biological Sciences* **361**, 1007-1022 (2006).
8. C. Brochier, P. Forterre, S. Gribaldo, Archaeal phylogeny based on proteins of the transcription and translation machineries: tackling the Methanopyrus kandleri paradox. *Genome biology* **5**, 1-12 (2004).
9. G. Borrel *et al.*, Phylogenomic data support a seventh order of methylotrophic methanogens and provide insights into the evolution of methanogenesis. *Genome biology and evolution* **5**, 1769-1780 (2013).
10. I. Vanwonterghem *et al.*, Methylotrophic methanogenesis discovered in the archaeal phylum Verstraetearchaeota. *Nature Microbiology* **1**, 16170 (2016).
11. L. J. McKay *et al.*, Co-occurring genomic capacity for anaerobic methane and dissimilatory sulfur metabolisms discovered in the Korarchaeota. *Nature Microbiology* **4**, 614-622 (2019).
12. Z.-S. Hua *et al.*, Insights into the ecological roles and evolution of methyl-coenzyme M reductase-containing hot spring Archaea. *Nature Communications* **10**, 4574 (2019).
13. G. Borrel *et al.*, Wide diversity of methane and short-chain alkane metabolisms in uncultured archaea. *Nature microbiology* **4**, 603-613 (2019).
14. Y. Wang *et al.*, A methylotrophic origin of methanogenesis and early divergence of anaerobic multicarbon alkane metabolism. *Science Advances* **7**, eabj1453 (2021).
15. B. A. Berghuis *et al.*, Hydrogenotrophic methanogenesis in archaeal phylum Verstraetearchaeota reveals the shared ancestry of all methanogens. *Proceedings of the National Academy of Sciences* **116**, 5037 (2019).
16. G. Borrel, P. S. Adam, S. Gribaldo, Methanogenesis and the Wood–Ljungdahl pathway: an ancient, versatile, and fragile association. *Genome biology and evolution* **8**, 1706-1711 (2016).
17. W. Martin, M. J. Russell, On the origin of biochemistry at an alkaline hydrothermal vent. *Philosophical Transactions of the Royal Society B: Biological Sciences* **362**, 1887-1926 (2007).
18. W. F. Martin, F. L. Sousa, Early microbial evolution: the age of anaerobes. *Cold Spring Harbor Perspectives in Biology* **8**, a018127 (2016).
19. F. L. Sousa *et al.*, Early bioenergetic evolution. *Philosophical Transactions of the Royal Society B: Biological Sciences* **368**, 20130088 (2013).
20. W. Martin, J. Baross, D. Kelley, M. J. Russell, Hydrothermal vents and the origin of life. *Nature Reviews Microbiology* **6**, 805-814 (2008).
21. K. Raymann, C. Brochier-Armanet, S. Gribaldo, The two-domain tree of life is linked to a new root for the Archaea. *Proceedings of the National Academy of Sciences* **112**, 6670-6675 (2015).
22. M. Aouad *et al.*, A divide-and-conquer phylogenomic approach based on character supermatrices resolves early steps in the evolution of the Archaea. *BMC Ecology and Evolution* **22**, 1 (2022).
23. P. S. Adam, G. Borrel, S. Gribaldo, Evolutionary history of carbon monoxide dehydrogenase/acetyl-CoA synthase, one of the oldest enzymatic complexes. *Proceedings of the National Academy of Sciences* **115**, E1166-E1173 (2018).
24. P. S. Adam, G. Borrel, S. Gribaldo, An archaeal origin of the Wood–Ljungdahl H4MPT branch and the emergence of bacterial methylotrophy. *Nature microbiology* **4**, 2155-2163 (2019).
25. P. N. Evans *et al.*, Methane metabolism in the archaeal phylum Bathyarchaeota revealed by genome-centric metagenomics. *Science* **350**, 434-438 (2015).
26. R. Laso-Pérez *et al.*, Thermophilic archaea activate butane via alkyl-coenzyme M formation. *Nature* **539**, 396-401 (2016).

27. R. Laso-Pérez *et al.*, Anaerobic Degradation of Non-Methane Alkanes by "Candidatus Methanoliparia" in Hydrocarbon Seeps of the Gulf of Mexico. *mBio* **10**, e01814-01819 (2019).
28. Y. Wang, G. Wegener, J. Hou, F. Wang, X. Xiao, Expanding anaerobic alkane metabolism in the domain of Archaea. *Nature Microbiology* **4**, 595-602 (2019).
29. Z. Zhou *et al.*, Non-syntrophic methanogenic hydrocarbon degradation by an archaeal species. *Nature* **601**, 257-262 (2022).
30. K. W. Seitz *et al.*, Asgard archaea capable of anaerobic hydrocarbon cycling. *Nature Communications* **10**, 1822 (2019).
31. J. A. Boyd *et al.*, Divergent methyl-coenzyme M reductase genes in a deep-subseafloor Archaeoglobi. *The ISME Journal* **13**, 1269-1279 (2019).
32. S.-C. Chen *et al.*, Anaerobic oxidation of ethane by archaea from a marine hydrocarbon seep. *Nature* **568**, 108-111 (2019).
33. C. J. Hahn *et al.*, "Candidatus Ethanoperedens," a Thermophilic Genus of Archaea Mediating the Anaerobic Oxidation of Ethane. *mBio* **11**, e00600-00620 (2020).
34. P. S. Adam, G. Borrel, C. Brochier-Armanet, S. Gribaldo, The growing tree of Archaea: new perspectives on their diversity, evolution and ecology. *The ISME Journal* **11**, 2407-2425 (2017).
35. M. Aouad *et al.*, Extreme halophilic archaea derive from two distinct methanogen Class II lineages. *Molecular Phylogenetics and Evolution* **127**, 46-54 (2018).
36. T. A. Williams *et al.*, Integrative modeling of gene and genome evolution roots the archaeal tree of life. *Proceedings of the National Academy of Sciences* **114**, E4602-E4611 (2017).
37. A. J. Probst *et al.*, Differential depth distribution of microbial function and putative symbionts through sediment-hosted aquifers in the deep terrestrial subsurface. *Nature microbiology* **3**, 328-336 (2018).
38. J. T. Bird, B. J. Baker, A. J. Probst, M. Podar, K. G. Lloyd, Culture independent genomic comparisons reveal environmental adaptations for Altiarchaeales. *Frontiers in microbiology* **7**, 1221 (2016).
39. D. Y. Sorokin *et al.*, Discovery of extremely halophilic, methyl-reducing euryarchaea provides insights into the evolutionary origin of methanogenesis. *Nature microbiology* **2**, 17081 (2017).
40. M. Aouad, G. Borrel, C. Brochier-Armanet, S. Gribaldo, Evolutionary placement of Methanonatronarchaeia. *Nature Microbiology* **4**, 558-559 (2019).
41. D. Y. Sorokin *et al.*, Reply to 'Evolutionary placement of Methanonatronarchaeia'. *Nature Microbiology* **4**, 560-561 (2019).
42. J. Martijn *et al.*, Hikarchaeia demonstrate an intermediate stage in the methanogen-to-halophile transition. *Nature Communications* **11**, 5490 (2020).
43. B. J. Baker *et al.*, Diversity, ecology and evolution of Archaea. *Nature Microbiology*, 1-14 (2020).
44. C. R. Woese, O. Kandler, M. L. Wheelis, Towards a natural system of organisms: proposal for the domains Archaea, Bacteria, and Eucarya. *Proceedings of the National Academy of Sciences* **87**, 4576-4579 (1990).
45. C. Petitjean, P. Deschamps, P. López-García, D. Moreira, Rooting the domain archaea by phylogenomic analysis supports the foundation of the new kingdom Proteoarchaeota. *Genome biology and evolution* **7**, 191-204 (2015).
46. T. Cavalier-Smith, E. E. Y. Chao, Multidomain ribosomal protein trees and the planctobacterial origin of neomura (eukaryotes, archaebacteria). *Protoplasma* **257**, 621-753 (2020).
47. E. R. Moody *et al.*, An estimate of the deepest branches of the tree of life from ancient vertically-evolving genes. *Elife* **11**, e66695 (2022).
48. L. A. Hug *et al.*, A new view of the tree of life. *Nature Microbiology* **1**, 16048 (2016).
49. C. Rinke *et al.*, Insights into the phylogeny and coding potential of microbial dark matter. *Nature* **499**, 431-437 (2013).
50. C. Rinke *et al.*, A standardized archaeal taxonomy for the Genome Taxonomy Database. *Nature Microbiology* **6**, 946-959 (2021).
51. K. S. Makarova, E. V. Koonin, Evolutionary and functional genomics of the Archaea. *Current opinion in microbiology* **8**, 586-594 (2005).
52. J. L. Weissman, S. Hou, J. A. Fuhrman, Estimating maximal microbial growth rates from cultures, metagenomes, and single cells via codon usage patterns. *Proceedings of the National Academy of Sciences* **118**, e2016810118 (2021).
